# Aneuploidy generates proteotoxic stress and DNA damage concurrently with p53-mediated post-mitotic apoptosis in SAC-impaired cells

**DOI:** 10.1038/ncomms8668

**Published:** 2015-07-06

**Authors:** Akihiro Ohashi, Momoko Ohori, Kenichi Iwai, Yusuke Nakayama, Tadahiro Nambu, Daisuke Morishita, Tomohiro Kawamoto, Maki Miyamoto, Takaharu Hirayama, Masanori Okaniwa, Hiroshi Banno, Tomoyasu Ishikawa, Hitoshi Kandori, Kentaro Iwata

**Affiliations:** 1 Oncology Drug Discovery Unit, Takeda Pharmaceutical Company Limited, 26-1, Muraoka-Higashi 2-chome, Fujisawa 251-8555, Japan; 2 Biomolecular Research Laboratories, Takeda Pharmaceutical Company Limited, 26-1, Muraoka-Higashi 2-chome, Fujisawa 251-8555, Japan; 3 DMPK Research Laboratories, Takeda Pharmaceutical Company Limited, 26-1, Muraoka-Higashi 2-chome, Fujisawa 251-8555, Japan; 4 Drug Safety Research Laboratories, Pharmaceutical Research Division, Takeda Pharmaceutical Company Limited, 26-1, Muraoka-Higashi 2-chome, Fujisawa 251-8555, Japan

## Abstract

The molecular mechanism responsible that determines cell fate after mitotic slippage is unclear. Here we investigate the post-mitotic effects of different mitotic aberrations—misaligned chromosomes produced by CENP-E inhibition and monopolar spindles resulting from Eg5 inhibition. Eg5 inhibition in cells with an impaired spindle assembly checkpoint (SAC) induces polyploidy through cytokinesis failure without a strong anti-proliferative effect. In contrast, CENP-E inhibition causes p53-mediated post-mitotic apoptosis triggered by chromosome missegregation. Pharmacological studies reveal that aneuploidy caused by the CENP-E inhibitor, Compound-A, in SAC-attenuated cells causes substantial proteotoxic stress and DNA damage. Polyploidy caused by the Eg5 inhibitor does not produce this effect. Furthermore, p53-mediated post-mitotic apoptosis is accompanied by aneuploidy-associated DNA damage response and unfolded protein response activation. Because Compound-A causes p53 accumulation and antitumour activity in an SAC-impaired xenograft model, CENP-E inhibitors could be potential anticancer drugs effective against SAC-impaired tumours.

## Abstract (web summary)

CENP-E regulates chromosome alignment during mitosis to distribute chromosomes equally into daughter cells. Here, the authors show that CENP-E inhibition causes p53-mediated post-mitotic apoptosis in tumours where the spindle assembly checkpoint is compromised, suggesting that CENP-E is a therapeutic target for these cancers.

## Graphical abstract

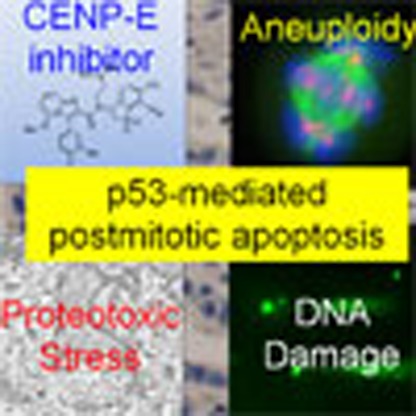

Accurate control of chromosome segregation during mitosis is crucial for genomic stability. Chromosome segregation during mitosis involves dynamic interactions between spindle microtubules and kinetochores. These interactions are required for bipolar attachment between kinetochores and microtubules and subsequent alignment of sister chromatids to the metaphase plate. To maintain fidelity during chromosome segregation, the spindle assembly checkpoint (SAC) mechanism regulates the proper attachment of microtubules to kinetochores and the tension between the kinetochores of sister chromatids^1^. SAC prevents premature sister chromatid separation until the kinetochores of each duplicated chromosome pair have achieved bipolar attachment to the mitotic spindle^2^. Components of SAC, such as Bub1, Bub3, BubR1, Mad1 Mad2 and Mps1, preferentially localize at the kinetochores of unaligned chromosomes, where they produce a diffusible ‘wait anaphase' signal^1^,^3^,^4^. This signal prevents the activation of the anaphase-promoting complex/cyclosome, degradation of target proteins and progression from metaphase to anaphase. Disruption of the kinetochore assembly, attachment of spindle microtubules or SAC activity often leads to chromosome missegregation or premature mitotic exit, a process known as mitotic slippage^5^, and consequently generates aneuploidy, a hallmark of many solid tumours^1^,^6^,^7^,^8^,^9^.

Antimitotic therapeutics such as taxanes or vinca alkaloids, which suppress microtubule dynamics in the mitotic spindle to activate SAC, are widely used in the clinical treatment of cancer^10^. Although the detailed functional mechanisms of these drugs remain unclear, prolonged mitotic arrest appears to be one of the central mechanisms underlying the anti-proliferative activity of these drugs. Sustained mitotic arrest can provide more opportunities for antimitotic drugs to induce apoptosis^11^. Thus, to rescue cancer cells from mitotic death, mitotic slippage by SAC downregulation could bypass prolonged mitotic arrest before activating the apoptotic pathway in lesions refractory to antimitotic inhibitors^5^,^12^,^13^,^14^,^15^,^16^. To overcome the difficulties in the treatment of tumours resistant to current antimitotic drugs, next-generation mitotic inhibitors are expected to be effective against SAC-impaired and SAC-intact tumours.

CENP-E and Eg5 are mitotic spindle motor proteins of the kinesin superfamily^17^. Eg5 regulates centrosome separation and bipolar mitotic spindle formation^18^,^19^,^20^. CENP-E is localized at the kinetochores of chromosomes^17^,^21^ and controls chromosome alignment during metaphase by capturing the microtubule plus-end at the kinetochore^22^,^23^,^24^. Loss of CENP-E function can result in misaligned chromosomes during metaphase, leading to SAC activation^23^,^24^,^25^,^26^,^27^,^28^,^29^,^30^. Furthermore, CENP-E acts as a signal-transducing linker for BubR1-dependent SAC signalling by capturing it at spindle microtubule kinetochores^29^, indicating that CENP-E regulates mitotic progression and checkpoint activity. Recently, small-molecule inhibitors targeting mitotic components such as CENP-E and Eg5 have been developed as cancer therapeutics^10^,^25^,^31^,^32^,^33^. In preclinical studies, these mitotic inhibitors suppressed the proliferation and increased the apoptosis of cancer cells via various mitotic aberrations, monopolar mitotic spindles, chromosome misalignment, lagging chromosomes, centrosome fragmentation and cytokinesis failure. However, the molecular relationships between mitotic aberrations and suppression of proliferation remain unclear.

In this study, we investigated the molecular mechanisms by which CENP-E and Eg5 inhibition suppress cancer cell proliferation. To analyse these processes, we used a chemical inhibitor of CENP-E, Compound-A (Cmpd-A), as well as short interfering RNA (siRNA)-based approaches. We reveal that under SAC-defective conditions, aneuploidy caused by CENP-E inhibition triggers p53 activation after mitotic slippage, resulting in a post-mitotic decrease in proliferation. Polyploidy caused by Eg5 inhibition does not produce this effect. Furthermore, we illustrate that aneuploidy-associated DNA damage response (DDR) and proteotoxic stress are accompanied by this post-mitotic p53 activation. These findings will help to elucidate the molecular mechanisms linking chromosome instability and anti-proliferation and promote translational research on mitotic inhibitors for cancer therapeutics.

## Results

### siCENP-E inhibits proliferation in a SAC-impaired condition

**Figure 1:**
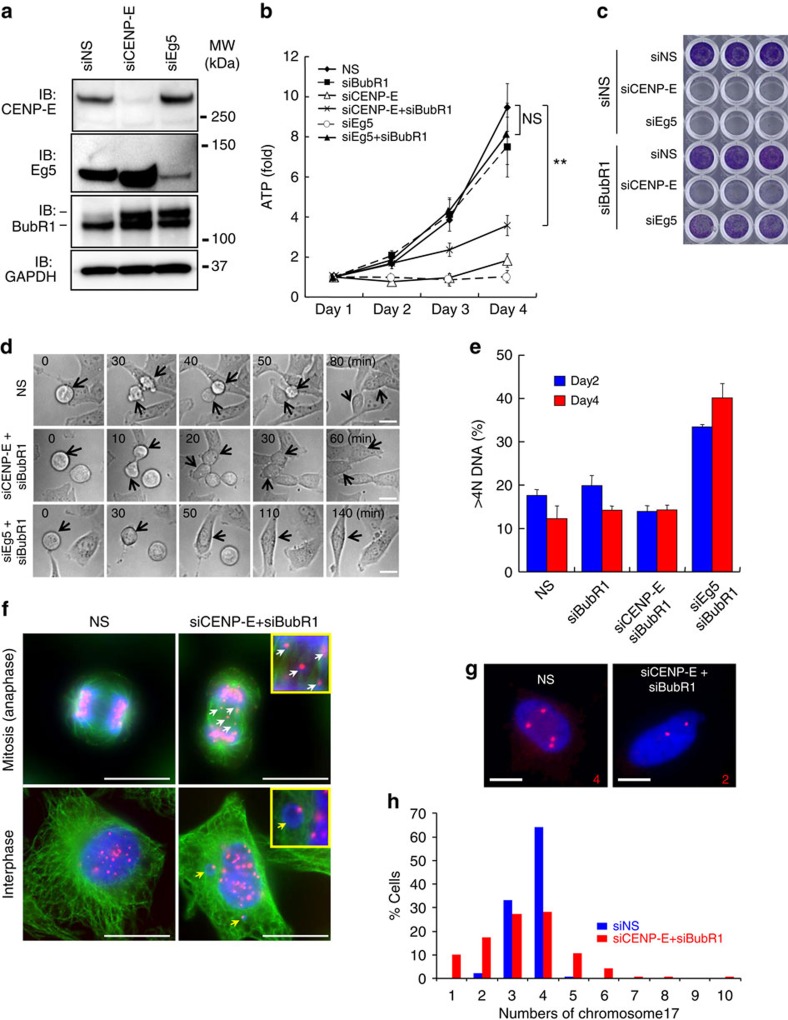
Post-mitotic growth inhibition and chromosome missegregation caused by siCENP-E and siBubR1. (**a**) Immunoblotting of BubR1 in HeLa cells transfected with siCENP-E and siEg5. The upper band in the BubR1 rectangle represents phosphorylated BubR1. (**b**) Anti-proliferative effects of siCENP-E in siBubR1-transfected HeLa cells. Cells transfected with the indicated siRNA were collected on days 1–4 after transfection. Relative ATP levels were calculated based on luminescence in comparison with the day-1 luminescence value (control). The line plots represent mean±s.d. (*n*=3). ATP levels on day 4 were statistically analysed using Student's *t*-test for comparison between siCENP-E+siBubR1- and siNS-transfected cells or between siEg5+siBubR1- and siNS-transfected cells. Differences were considered significant at *P*≤0.05 (*) and *P*≤0.01 (**). (**c**) Representative images of crystal violet staining in HeLa cells transfected with the indicated siRNAs. Cells were collected on day 3 after transfection for crystal violet staining. (**d**) Time-lapse microscopy of HeLa cells transfected with siNS (upper row), siCENP-E+siBubR1 (middle row) or siEg5+siBubR1 (lower row). Time point *t*=0 indicates the onset of mitosis. Arrows indicate cells undergoing mitosis. White bars indicate 20 μm. (**e**) Quantification of the >4N DNA population was determined by FACS analysis on days 2 and 4 after siRNA transfection. Data are presented as mean±s.d. (*n*=3). (**f**) Micronuclei and lagging chromosomes in siCENP-E- and siBubR1-transfected HeLa cells. Green, red and blue signals indicate α-tubulin, CENP-B and DAPI, respectively. White and yellow arrows denote the micronuclei and lagging chromosomes, respectively. White bars indicate 20 μm. (**g,h**) Fluorescence *in situ* hybridization (FISH) analysis using centromeric probes. FISH analysis using centromeric probes for Chr-17 was performed 48 h after siRNA transfection (red, Chr-17 centromeres; blue, DNA). Representative FISH images of cells transfected with siNS or siCENP-E+siBubR1 (**g**). White bars indicate 10 μm. The graph shows quantitative FISH analysis (blue, siNS-transfected cells (*n*=139); red, siCENP-E+siBubR1-transfected cells (*n*=139); **h**).

To evaluate the potential of CENP-E as a cancer therapeutic target, we investigated the molecular mechanisms by which CENP-E regulates cancer cell proliferation using siRNA-based approaches. Eg5 was used as a control because the loss of Eg5 function causes monopolar spindles and subsequent apoptosis during prolonged mitotic arrest^18^,^19^,^34^ (Supplementary Fig. 1a–d). siRNAs of both CENP-E (siCENP-E) and Eg5 (siEg5) induced BubR1 phosphorylation, which activates SAC (Fig. 1a). BubR1 knockdown (siBubR1) released both siCENP-E- and siEg5-transfected cells from prolonged mitotic arrest (Supplementary Fig. 1d), demonstrating that BubR1 monitored aberrant chromosome dynamics caused by siCENP-E or siEg5 and triggered SAC activation. We next determined whether SAC attenuation by siBubR1 could rescue siCENP-E- or siEg5-transfected cells from apoptosis and recover their viability. siCENP-E or siEg5 transfection had a potent anti-proliferative effect and caused death in HeLa cells (Fig. 1b (open triangle) and (open circle) and Supplementary Fig. 1a,b,e) within 72 h after transfection. After double knockdown of Eg5 and BubR1 (siEg5+siBubR1), cell viability was significantly restored with release from prolonged mitotic arrest (Fig. 1b (closed triangle) and Supplementary Figs 1d,e, 2 and 3). Conversely, the double knockdown of CENP-E and BubR1 (siCENP-E+siBubR1) did not restore cell viability (Fig. 1b and Supplementary Figs 2 and 4). In siCENP-E+siBubR1 transfection experiments, the cells exited prolonged mitotic arrest (Supplementary Fig. 1d), and cell viability was rescued on day 2 (Fig. 1b). This result showed that the post-mitotic effects elicited anti-proliferation in these cells 24 h or later after mitotic death was bypassed. Crystal violet staining also confirmed that siBubR1 restored viability in siEg5-transfected but not in siCENP-E-transfected cells (Fig. 1c and Supplementary Fig. 5a,b). These data demonstrate that although SAC attenuation can release both siCENP-E- and siEg5-transfected cells from cell death during prolonged mitotic arrest, the post-mitotic effect suppresses proliferation in siCENP-E+siBubR1-transfected but not in siEg5+siBubR1-transfected cells.

**Figure 2:**
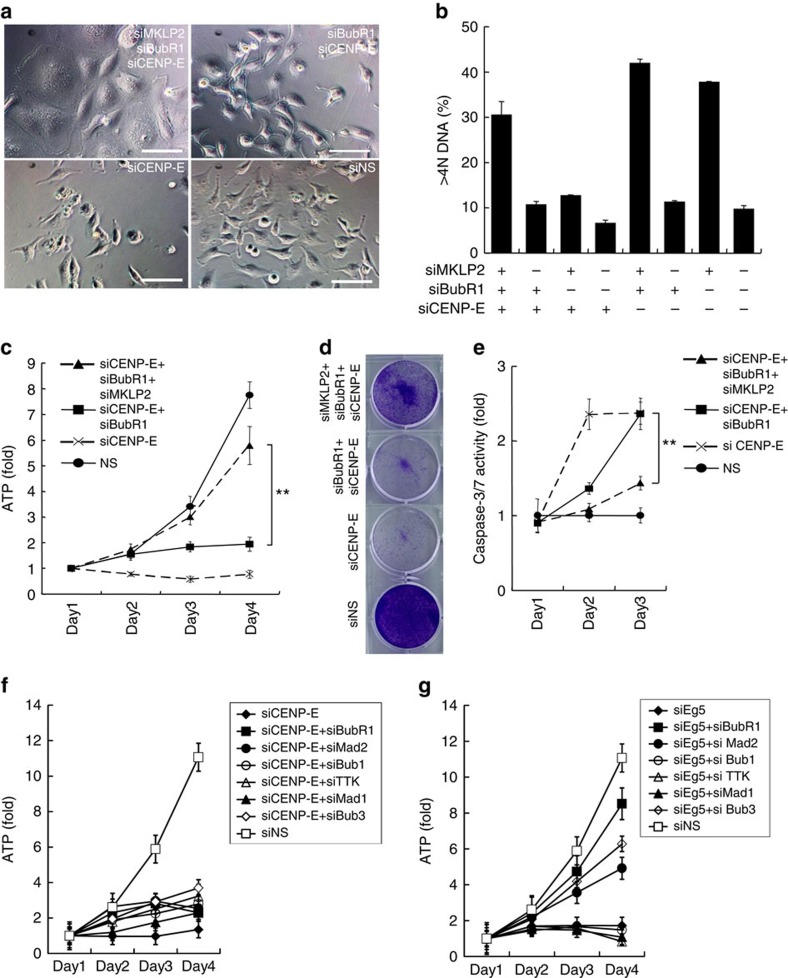
Failure of cytokinesis restores cell viability in siCENP-E+siBubR1-transfected cells. (**a**) Phase-contrast microscopy images of CENP-E, BubR1 and MKLP2 triple-knockdown cells. Images were acquired 3 days after siRNA transfection. White bars indicate 100 μm. (**b**) The >4N DNA population was quantified by FACS analysis 48 h after siRNA transfection. Data are presented as mean±s.d. (*n*=3). (**c**) Proliferative effect of siMKLP2 in siCENP-E+siBubR1-transfected HeLa cells. The cells were transfected with the indicated siRNAs. Cell growth was evaluated as indicated in Fig. 1b. The line plots represent mean±s.d. (*n*=3). ATP levels on day 4 were statistically analysed using Student's *t*-test for comparisons between siCENP-E+siBubR1+siMKLP2- and siCENP-E+siBubR1-transfected cells. Differences were considered significant at *P*≤0.05 (*) and *P*≤0.01 (**). (**d**) Representative images of crystal violet staining in HeLa cells transfected with the indicated siRNAs. Cells were collected on day 3 after transfection. (**e**) Apoptotic effect of siMKLP2 in siCENP-E+siBubR1-transfected HeLa cells. Relative caspase-3/7 activities were calculated based on the luminescence in comparison with the day-1 luminescence value (control). The line plots represent mean±s.d. (*n*=3). Caspase-3/7 activities on day 3 were statistically analysed using Student's *t*-test for comparison between siCENP-E+siBubR1+siMKLP2- and siCENP-E+siBubR1-transfected cells. Differences were considered significant at *P*≤0.05 (*) and *P*≤0.01 (**). (**f**,**g**) Proliferative effect of the knockdown of SAC-associated genes in siCENP-E- or siEg5-transfected HeLa cells. The cells were transfected with siCENP-E (**e**) or siEg5 (**f**) in combination with the indicated siRNAs. Cell growth was evaluated as indicated in Fig. 1b. The line plots represent mean±s.d. (*n*=3).

Time-lapse microscopy revealed that siCENP-E+siBubR1 transfection allowed the cells to undergo cytokinesis, as did non-silencing siRNA (Fig. 1d). However, the presence of siEg5+siBubR1 induced cytokinesis failure, generating polyploid cells. Polyploidy (>4N DNA) increased in siEg5+siBubR1-transfected cells but not in siCENP-E+siBubR1-transfected cells (Fig. 1e). Because Eg5 inhibition causes monopolar spindle formation, SAC attenuation by siBubR1 causes polyploidy in siEg5-transfected cells through cytokinesis failure. Conversely, in siCENP-E+siBubR1-transfected cells, lagging chromosomes or micronuclei, the hallmarks of chromosome missegregation, were observed frequently (Fig. 1f). To assess chromosome missegregation directly, fluorescence *in situ* hybridization was performed with Cy3-labelled probes for chromosome 17 (Chr-17) centromeres (Fig. 1g,h). In HeLa cells, three to four copies of Chr-17 were found in 97.1% (135/139 cells) of the control cells but only 55.4% (77/139 cells) of siCENP-E+siBubR1-transfected cells. Furthermore, the range of Chr-17 copy numbers in siCENP-E+siBubR1-transfected cells was wider (1–10 chromosomes) than that in the control cells. To investigate the effect of asymmetric chromosome segregation on proliferation, we induced cytokinesis failure in siCENP-E+siBubR1-transfected cells. Cytokinesis failure produces polyploid daughter cells with twofold more chromosomes than their parental cells. Therefore, it does not result in aneuploid daughter cells with copy-number variations for each chromosome caused by asymmetric chromosome segregation. Mitotic kinesin-like protein 2 (MKLP2), also known as kinesin superfamily protein 20A (KIF20A)^17^, is involved in the localization of several mitotic proteins at the central spindle where they regulate cytokinesis^35^. Knockdown of MKLP2 (siMKPL2) induced cytomegalic phenotypes in siCENP-E+siBubR1-transfected cells. Among the cells with triple knockdown of CENP-E, BubR1 and MKLP2 (siCENP-E+siBubR1+siMKLP2), the population with >4N DNA cells was significantly larger than that among siCENP-E+siBubR1-transfected cells (Fig. 2a,b and Supplementary Fig. 6a–c). In the polyploid cells induced by siCENP-E+siBubR1+siMKPL2, similar to those induced by siEg5+siBubR1, cell viability was restored to a significantly higher level than that in the aneuploid cells induced by siCENP-E+siBubR1 (Fig. 2c,d and Supplementary Fig. 6d,e). Supporting their proliferative recovery, caspase-3/7 activity was significantly lower in siCENP-E+siBubR1+siMKPL2-transfected cells than in siCENP-E+siBubR1-transfected cells (Fig. 2e). We also determined the effect of other SAC-associated genes on cell viability in siCENP-E-transfected cells compared with siEg5-transfected cells. Knockdown of Mad2 and Bub3 significantly restored the viability of siEg5-transfected cells. The viability of siCENP-E-transfected cells was unaffected by the knockdown of any SAC-associated gene (Fig. 2f,g and Supplementary Fig. 7a–f). In summary, CENP-E inhibition coupled with SAC attenuation increases asymmetric chromosome segregation, which is responsible for the post-mitotic anti-proliferative effect.

### Aneuploidy upregulates the p53 pathway in SAC-impaired cells

**Figure 3:**
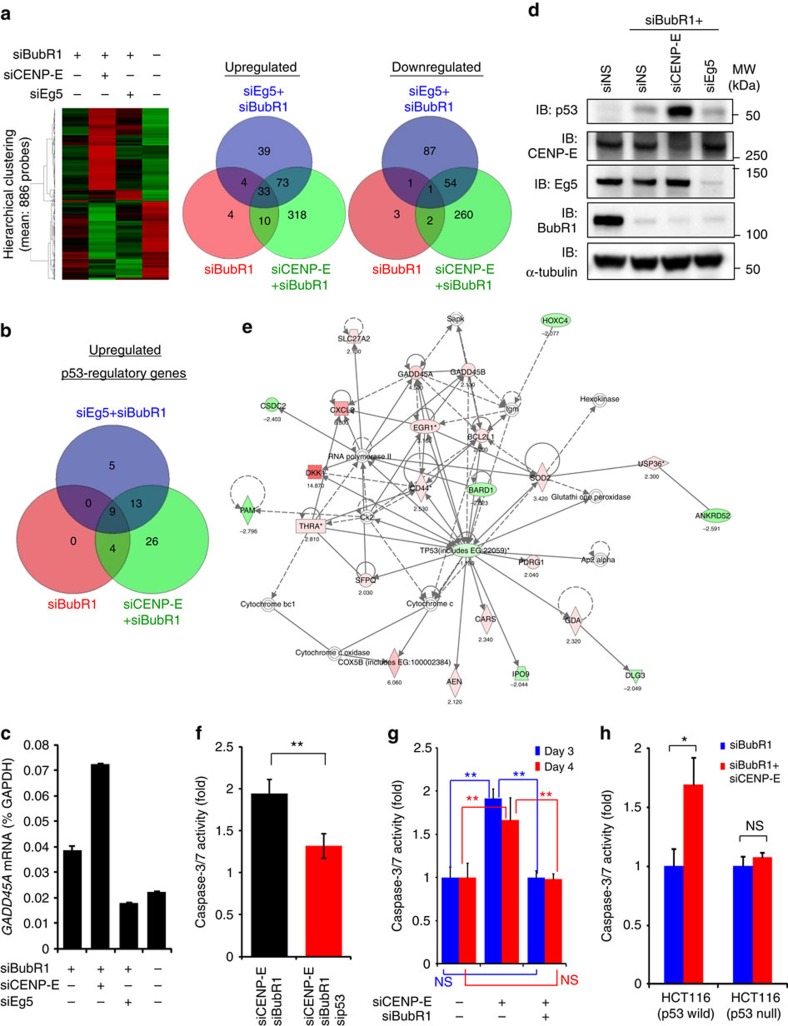
Comprehensive gene expression analysis in siCENP-E+siBubR1-transfected cells. (**a**) Microarray analysis of the transcriptome of HeLa cells transfected with the indicated siRNAs. (Left) Hierarchical cluster analysis of 886 transcript expression profiles. Each row represents a single transcript. Red and green: relatively high and low expression, respectively. (Right) Venn diagrams. ‘Upregulated' and ‘Downregulated': >2-fold increase and >2-fold decrease, respectively, in the expression of the indicated transcripts compared with siNS-transfected cells. (**b**) Venn diagram-related to p53 pathways. ‘Upregulated': >2-fold increase in the levels of 343 p53-regulated transcripts compared with siNS-transfected cells. (**c**) Quantitative RT–PCR analysis of GAD45A in HeLa cells 72 h after transfection. GAD45A expression ratios were quantified using GAPDH expression. Data are presented as mean±s.d. (*n*=3). (**d**) Protein expression of p53 in siCENP-E+siBubR1-transfected cells on day 3 after transfection. (**e**) Pathway analysis. The network was generated by Ingenuity Pathway Analysis (Ingenuity Systems, http://www.ingenuity.com) using p53 and 434 upregulated and 317 downregulated genes in siCENP-E+siBubR1-transfected cells. (**f**) Effect of p53 on caspase-3/7 activity in siCENP-E+siBubR1-transfected cells. Black and red bars: siCENP-E+siBubR1-transfected and siCENP-E+siBubR1+sip53-transfected cells, respectively. Cells were collected on day 3 after transfection. Caspase-3/7 activities were evaluated as indicated in Fig. 2e. Data are presented as mean±s.d. (*n*=3). Student's *t*-test was used to compare siCENP-E+siBubR1+sip53- and siCENP-E+siBubR1-transfected cells. Differences were considered significant at *P*≤0.05 (*) and *P*≤0.01 (**). (**g**) Effect of p53 on caspase-3/7 activity in siCENP-E+siBubR1-transfected SK-BR3 cells. Cells were collected on days 3 (blue) and 4 (red) after transfection. Caspase-3/7 activities were evaluated as indicated in Fig. 2e. Data are presented as mean±s.d. (*n*=3). Statistical analysis was performed using Student's *t*-test. Differences were considered significant at *P*≤0.05 (*) and *P*≤0.01 (**). (**h**) Caspase-3/7 activation by siCENP-E+siBubR1 in p53-wild-type and p53-knockout HCT116 cells on day 3 after transfection. Caspase-3/7 activities were evaluated as indicated in Fig. 2e. Data are presented as mean±s.d. (*n*=3). Statistical analysis was performed using Student's *t*-test. Differences were considered significant at *P*≤0.05 (*) and *P*≤0.01 (**).

To determine which signalling pathways contribute to the post-mitotic anti-proliferative effect of asymmetric chromosome segregation, comprehensive gene expression analysis was performed by microarray analysis (Fig. 3a and Supplementary Fig. 8a). Because the single knockdown of CENP-E or Eg5 caused substantial cell death within 3 days after transfection (Fig. 1b and Supplementary Figs 1–4), cell lysates from these cells could not be used in the microarray, reverse transcription–PCR (RT–PCR) or immunoblotting experiments. Thus, siNS-transfected cells were used as a control. Compared with the control cells, 52 of the 343 p53-regulated transcripts were upregulated in siCENP-E+siBubR1-transfected cells, whereas 27 and 13 transcripts were upregulated in siEg5+siBubR1- and siBubR1-transfected cells, respectively (Fig. 3b and Supplementary Fig. 8b,c). RT–PCR analysis also confirmed that GADD45A expression increased in siCENP-E+siBubR1-transfected cells (Fig. 3c). Although HeLa cells possess wild-type *p53*, human papillomavirus (HPV)-encoded E6/E7 genes downregulate the expression of the p53 protein. However, p53 expression was increased in siCENP-E+siBubR1-transfected HeLa cells, consistent with the microarray data (Fig. 3d). We also confirmed the upregulation of p53 by siCENP-E+siBubR1 in the HPV-negative but p53-wild-type cancer cell lines HCT116 and H460 (Supplementary Fig. 8d–f). This result indicates that p53 accumulation induced by siCENP-E+siBubR1 is independent of the HPV-associated p53 degradation machinery. Thus, in our model, HPV-encoded E6/E7 had little effect on the detected p53 accumulation. We mainly used HeLa cells in the subsequent experiments because of their technical availability for various assays. Pathway analysis using 434 upregulated genes, 317 downregulated genes and p53 itself also confirmed the central role of the p53 network in cell death and survival (Fig. 3e). Because p53 and caspase-3/7 expression was elevated concurrently (Fig. 2c,e and Supplementary Fig. 8g), we induced additional p53 knockdown in siCENP-E+siBubR1-transfected cells to examine the relationship between p53 and caspase-3/7 elevation. Compared with the control cells, a twofold increase in caspase-3/7 activity was detected in siCENP-E+siBubR1-transfected cells. Caspase-3/7 activity in siCENP-E+siBubR1+sip53-transfected cells was 1.3-fold lower (Fig. 3f and Supplementary Fig. 8h). Conversely, in the p53-defective cell line SK-BR3 and p53-knockout HCT116 cells, siCENP-E+siBubR1 did not induce caspase-3/7 activation (Fig. 3g,h and Supplementary Fig. 8i). CENP-E knockdown alone activated caspase-3/7 in SK-BR3 and HeLa cells (Figs 2e and 3g), demonstrating that caspase-3/7 activation during mitotic death in cells with intact SAC is regulated in a p53-independent manner. These data suggest that chromosome missegregation by CENP-E inhibition coupled with SAC attenuation activates p53 pathways, causing a post-mitotic anti-proliferative effect following mitotic slippage.

### Aneuploidy generates replication stress-mediated DSBs

**Figure 4:**
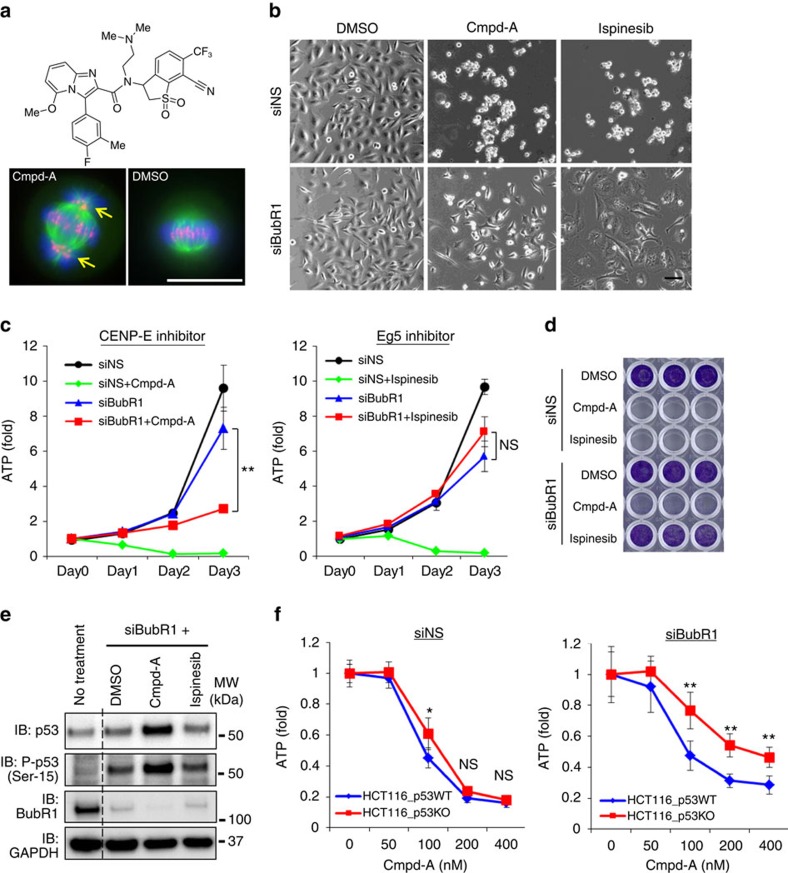
Pharmacological inhibition of CENP-E causes p53-associated suppression of proliferation in SAC-impaired cells but not in SAC-intact cells. (**a**) Chemical structure of Cmpd-A (Upper) and misaligned chromosomes in HeLa cells with Cmpd-A treatment (Lower). Green, red and blue signals indicate α-tubulin, CENP-B and DAPI, respectively. Yellow arrows denote the misaligned chromosomes. White bars indicate 20 μm. (**b**) Phase-contrast microscopy images of siNS- and siBubR1-transfected cells treated with DMSO, Cmpd-A and ispinesib. Twenty-four hours after siRNA treatment, the cells were treated with DMSO, Cmpd-A (200 nM) or ispinesib (10 nM). Images were acquired 48 h after drug treatment. The black bar indicates 100 μm. (**c**) Anti-proliferative effect of Cmpd-A and ispinesib in siBubR1-transfected HeLa cells. Twenty-four hours after siRNA treatment, the cells were treated with DMSO, Cmpd-A (200 nM) or ispinesib (10 nM) (day 0). Cell growth was evaluated as indicated in Fig. 1b. The line plots represent mean±s.d. (*n*=3). ATP levels on day 3 were statistically analysed using Student's *t*-test for comparison between siBubR1+Cmpd-A- and siBubR1-transfected cells and between siBubR1+ispinesib- and siBubR1-transfected cells. Differences were considered significant at *P*≤0.05 (*) and *P*≤0.01 (**). (**d**) Representative images of crystal violet staining in siNS- and siBubR1-transfected cells treated with DMSO, Cmpd-A and ispinesib. Twenty-four hours after siRNA treatment, the cells were treated with DMSO, Cmpd-A (200 nM) or ispinesib (10 nM). Cells were collected 5 days after drug treatment for crystal violet staining. (**e**) Immunoblotting of p53 and phospho-p53 in siBubR1-transfected HeLa cells treated with Cmpd-A and ispinesib. Cells were collected 48 h after drug treatment. (**f**) Anti-proliferative effect of Cmpd-A with or without siBubR1 in p53-wild-type and p53-knockout HCT116 cells. Twenty-four hours after siRNA treatment, the cells were treated with Cmpd-A at the indicated concentrations. Cells were collected on day 3 after drug treatment for the ATP assay. Relative ATP levels were calculated based on luminescence in comparison with the luminescence value for 0 nM treatment. The line plots represent mean±s.d. (*n*=3). Statistical analysis was performed using Student's *t*-test. Differences were considered significant at *P*≤0.05 (*) and *P*≤0.01 (**).

A novel small-molecule inhibitor of CENP-E, Cmpd-A ((+)-*N*-[7-cyano-1,1-dioxido-6-(trifluoromethyl)-2,3-dihydro-1-benzothiophen-3-yl]-*N*-[2-(dimethylamino)ethyl]-3-(4-fluoro-3-methylphenyl)-5-methoxyimidazo[1,2-*a*]pyridine-2-carboxamide), was developed (Fig. 4a, Supplementary Methods). The seed compounds were discovered by high-throughput chemical library screening with ATPase assays. Cmpd-A represents a chemically optimized molecule with the seed compounds. Cmpd-A inhibits the ATPase activity of the CENP-E-motor domain, with an IC_50_ value of 2.2 nM. It does not inhibit Eg5 or KHC kinesins or any of the other 36 tested kinases (Supplementary Figs 9 and 10). Treatment of HeLa cells with Cmpd-A caused chromosome misalignment, pHH3 accumulation, potent growth inhibition and cell death within 48 h (Fig. 4a–d and Supplementary Fig. 11a, GI_50_=80 nM). Given that CENP-E also functions as a signal-transducing linker in SAC signalling^29^, inhibition of this enzyme by Cmpd-A may induce more prolonged mitotic arrest than siCENP-E alone (Supplementary Fig. 11b,c). We analysed the effect of the pharmacological inhibition of CENP-E with Cmpd-A in comparison with the chemical inhibition of Eg5 by ispinesib. Supporting the results of our siRNA studies, treatment with Cmpd-A, but not with ispinesib, had a potent anti-proliferative effect in siBubR1-transfected HeLa cells (Fig. 4c,d and Supplementary Fig. 11d–f). Whereas Cmpd-A or ispinesib monotherapy caused substantial cell death within 48 h (Fig. 4b,c and Supplementary Fig. 11a), p53 accumulation was not detected in these cells. These results indicate that unlike aneuploidy-associated post-mitotic cell death, mitotic cell death caused by CENP-E or Eg5 inhibition is independent of p53. Samples taken 72 h after these single-agent treatments could not be used in further assays because of the high levels of cell death (Fig. 4b–d). Thus, siNS+dimethylsulphoxide (DMSO)-treated cells were used as a control. In agreement with the results of our siRNA-based studies, substantial p53 accumulation was detected in siBubR1-transfected cells treated with Cmpd-A (siBubR1+Cmpd-A; Fig. 4e). This was not observed in siBubR1-transfected cells treated with ispinesib (siBubR1+ispinesib). To assess the effects of p53 on the anti-proliferative activity of Cmpd-A, we conducted cell viability assays in p53 isogenic HCT116 cell lines. Cmpd-A treatment without siBubR1 had similar anti-proliferative effects in p53-wild-type and p53-knockout HCT116 cells, whereas Cmpd-A treatment with siBubR1 was more effective in p53-wild-type cells than in p53-knockout cells (Fig. 4f). These results also support the assumption that p53 contributes to aneuploidy-associated anti-proliferative effects after mitotic slippage.

**Figure 5:**
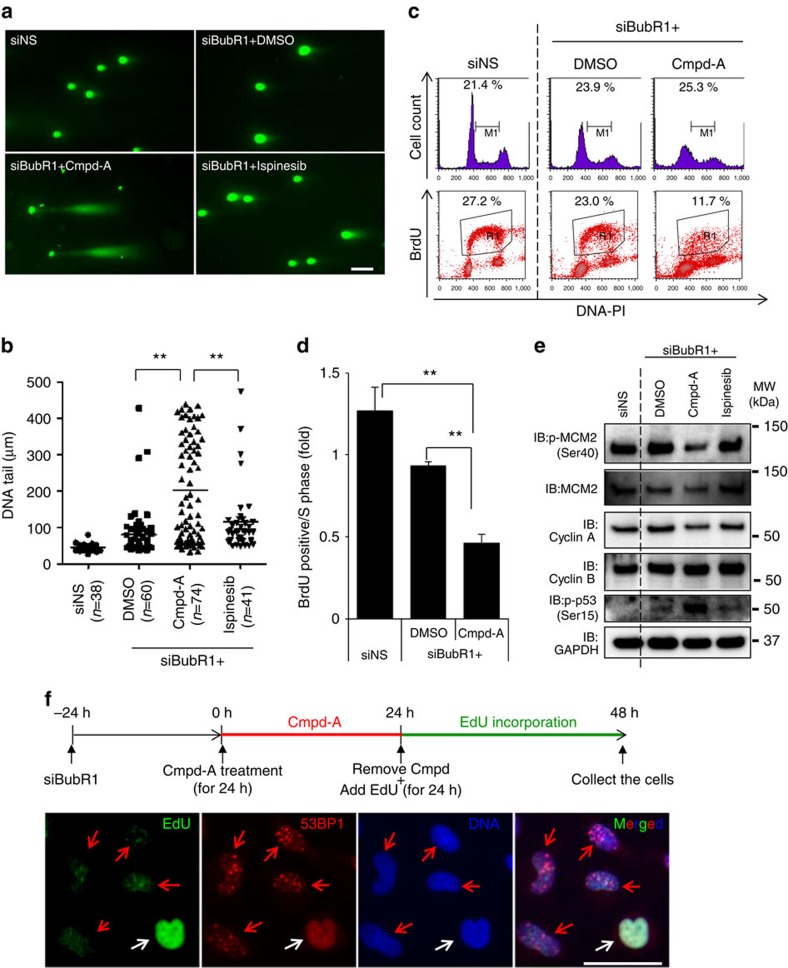
CENP-E inhibition causes replication stress-mediated DSBs under SAC-impaired conditions. (**a**) Representative images of the neutral comet assay in siBubR1+Cmpd-A-treated cells. Twenty-four hours after siRNA treatment, the cells were treated with DMSO, Cmpd-A (200 nM) or ispinesib (10 nM) for 72 h. The white bar indicates 100 μm. (**b**) Quantification of DNA tails in the neutral comet assay. The length of DNA tails in microscopy images was quantified by AxioVision. Statistical analysis was performed using Student's *t*-test. Differences were considered significant at *P*≤0.01 (**). (**c**) Cell cycle analysis in siBubR1+Cmpd-A-treated cells. Twenty-four hours after siRNA treatment, the cells were treated with DMSO, Cmpd-A (200 nM) or ispinesib (10 nM) for 72 h. BrdU was incorporated into the drug-treated cells for 15 min, and then the cells were collected for FACS analysis. Representative data are shown. (**d**) Replication activity in the S phase in siBubR1+Cmpd-A-treated cells. BrdU-positive cells (R1) were normalized to the S phase cells (M1). Data are presented as mean±s.d. (*n*=3).Statistical analysis was performed using Student's *t*-test. Differences were considered significant at *P*≤0.05 (*) and *P*≤0.01 (**). (**e**) Immunoblotting of replication-regulating and G2 phase proteins in siBubR1+Cmpd-A-treated cells. Cells were collected 72 h after drug treatment. (**f**) Correlation between 53BP1 foci formation and EdU incorporation. Schematics of the experiments are shown (above the panels). Red and white arrows indicate 53BP1 foci-positive and 53BP1 foci-negative cells, respectively. The white bar indicates 50 μm.

Similar to siCENP-E+siBubR1-transfected cells (Supplementary Figs 8e and 12a), we detected p53 phosphorylation at Ser-15 in siBubR1+Cmpd-A-treated cells (Fig. 4e). The increase in γH2AX (a marker of DNA double-strand breaks (DSBs)) levels also paralleled p53 accumulation in these cells (Supplementary Fig. 12b,c), suggesting that DSBs were generated in these cells. More directly, the neutral comet assay revealed that DNA tails, which indicate DSBs, were significantly longer in siBubR1+Cmpd-A-treated cells (Fig. 5a,b and Supplementary Fig. 12d). Furthermore, inhibitors of ATM (Ku55933) and ATR (VE-821)^36^,^37^ suppressed p53 phosphorylation and p21 expression (Supplementary Fig. 12e). To summarize, chromosome missegregation caused by CENP-E inhibition and SAC attenuation generated DSBs, and the activated DDR contributed to p53 phosphorylation by ATM and ATR. We next determined whether DSBs in aneuploid cells are generated during the S phase. Fluorescence-activated cell sorting (FACS) analysis revealed that BrdU-positive cells (in the S phase) were significantly reduced by siBubR1+Cmpd-A treatment, whereas neither G1 nor G2 arrest was observed (Fig. 5c,d and Supplementary Fig. 13a,b). Conversely, BrdU incorporation in siBubR1+ispinesib-treated polyploid cells did not significantly decrease compared with that in siBubR1+DMSO-treated cells (Supplementary Fig. 13a,b). Immunoblotting also revealed that the levels of phosphorylated MCM2 at Ser40, MCM2 and Cyclin A, which are DNA replication regulatory proteins, were decreased in siBubR1+Cmpd-A-treated cells (Fig. 5e), whereas the level of the G2 phase protein Cyclin B was not reduced. To determine the correlation between impairment of DNA replication and DSB accumulation, we performed immunofluorescence 5-ethynyl-2′-deoxyuridine (EdU) incorporation assays for 53BP1. As DSBs were generated mainly between 24 and 48 h after Cmpd-A treatment (Supplementary Fig. 12c), siBubR1-transfected HeLa cells were first treated with Cmpd-A for 24 h. Then, EdU was added, and the cells were incubated for the next 24 h without Cmpd-A (Fig. 5f). Immunofluorescence analysis revealed 53BP1 foci in EdU-negative cells (Fig. 5f, red arrows) but not in EdU-positive cells (Fig. 5f, white arrows). As the caspase inhibitor ZVAD-FMK had little effect on 53BP1 foci (Supplementary Fig. 13c), DSBs detected in these cells were not the consequence of apoptosis-inducing DNA breaks. In summary, chromosome missegregation accelerated by siBubR1+Cmpd-A treatment reduces replication activity during the S phase; this replication stress appears to be responsible for the generation of aneuploidy-associated DSBs.

### Aneuploidy induces proteotoxic stress and UPR activation

**Figure 6:**
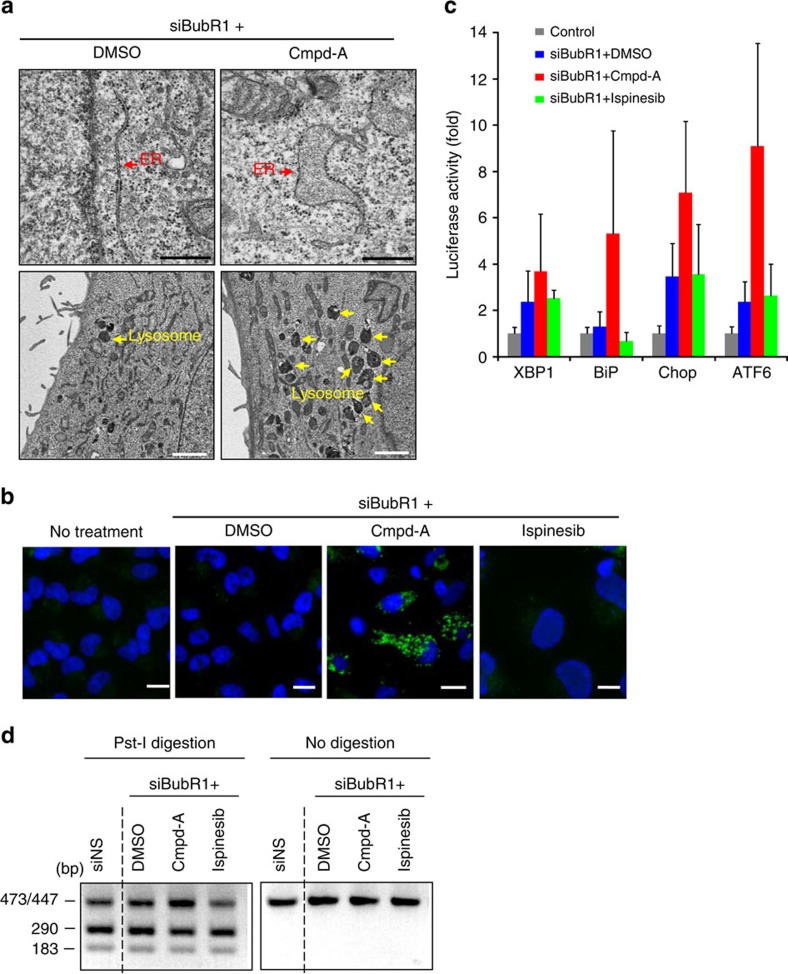
Aneuploidy-associated proteotoxic stress is accompanied by p53 accumulation after mitotic slippage. (**a**) Electron microscopy images of the ER (upper panels, red arrows) and lysosome (lower panels, yellow arrows) in siBubR1+Cmpd-A-treated HeLa cells. Twenty-four hours after siRNA treatment, the cells were treated with Cmpd-A (200 nM) or DMSO for 48 h. Black and white bars indicate 500 nm and 2 μm, respectively. (**b**) Immunofluorescence of LC3B in siBubR1+DMSO-, siBubR1+Cmpd-A- and siBubR1+ispinesib-treated HeLa cells. White bars indicate 20 μm. (**c**) Transcriptional reporter activities of XBP1, BiP, Chop and ATF6 in siBubR1+Cmpd-A-treated HeLa cells. Transfection with siRNA was performed 24 h before drug treatment, and the cells were treated with DMSO, Cmpd-A (200 nM) or ispinesib (10 nM) for 72 h. Relative luciferase activities were calculated based on the luminescence values in comparison with siNS-transfected cells. Data are presented as mean±s.d. (*n*=3). (**d**) XBP1 splicing assay. cDNA was treated with (left) or without (right) Pst-I. The undigested upper band (473/447 bp) and the digested lower bands (290 and 183 bp) represent spliced and unspliced XBP1, respectively.

Recent studies have revealed that the excess of proteins produced by additional chromosomes disturbs the protein quality control systems in aneuploid yeast cells, leading to growth deficiency^38^,^39^. We investigated whether aneuploidy-associated proteotoxic stress can be observed in siBubR1+Cmpd-A-treated cells. Electron microscopy analysis detected the expansion of the endoplasmic reticulum (ER; Fig. 6a, upper panels) and increased numbers of lysosomes (Fig. 6a, lower panels) in siBubR1+Cmpd-A-treated HeLa cells relative to controls. Furthermore, accumulation of insoluble sequestosome 1 (p62), p62 aggresome formation^40^,^41^ and autophagic vesicle-associated form of light-chain 3B (LC3B-II) were also prominently detected in siBubR1+Cmpd-A-treated cells but not in siBubR1+ispinesib- or siBubR1+DMSO-treated cells (Fig. 6b and Supplementary Fig. 14a–c). Although the effects of Cmpd-A were relatively mild, siBubR1+Cmpd-A treatment also caused p53 and LC3B induction in MCF10A untransformed mammary gland epithelial cells (Supplementary Fig. 15a–c). These results illustrate that aneuploidy-mediated protein homeostasis imbalance produces aggresomes of misfolded proteins. Then, autophagy is triggered to alleviate aneuploidy-associated proteotoxic stress by degrading abnormal or potentially toxic proteins^42^,^43^. We next established reporter assay systems for unfolded protein response (UPR) markers, namely the ER luminal chaperone BiP, ER membrane-associated protein ATF6 and UPR mediators XBP1 and Chop^44^. The reporter assays revealed increased levels of all tested markers in siBubR1+Cmpd-A-treated cells (Fig. 6c, red bars) but not in siBubR1+ispinesib-treated cells (Fig. 6c, green bars). An XBP1 splicing assay^45^ confirmed UPR activation in these cells (Fig. 6d and Supplementary Fig. 16a). Similar to the results obtained with Cmpd-A or ispinesib, siCENP-E+siBubR1-transfected cells, but not siEg5+siBubR1-transfected cells, also exhibited 53BP1 foci, LC3B-II formation, p62 accumulation, UPR reporter activation and XBP1 splicing (Supplementary Fig. 14b–e).

**Figure 7:**
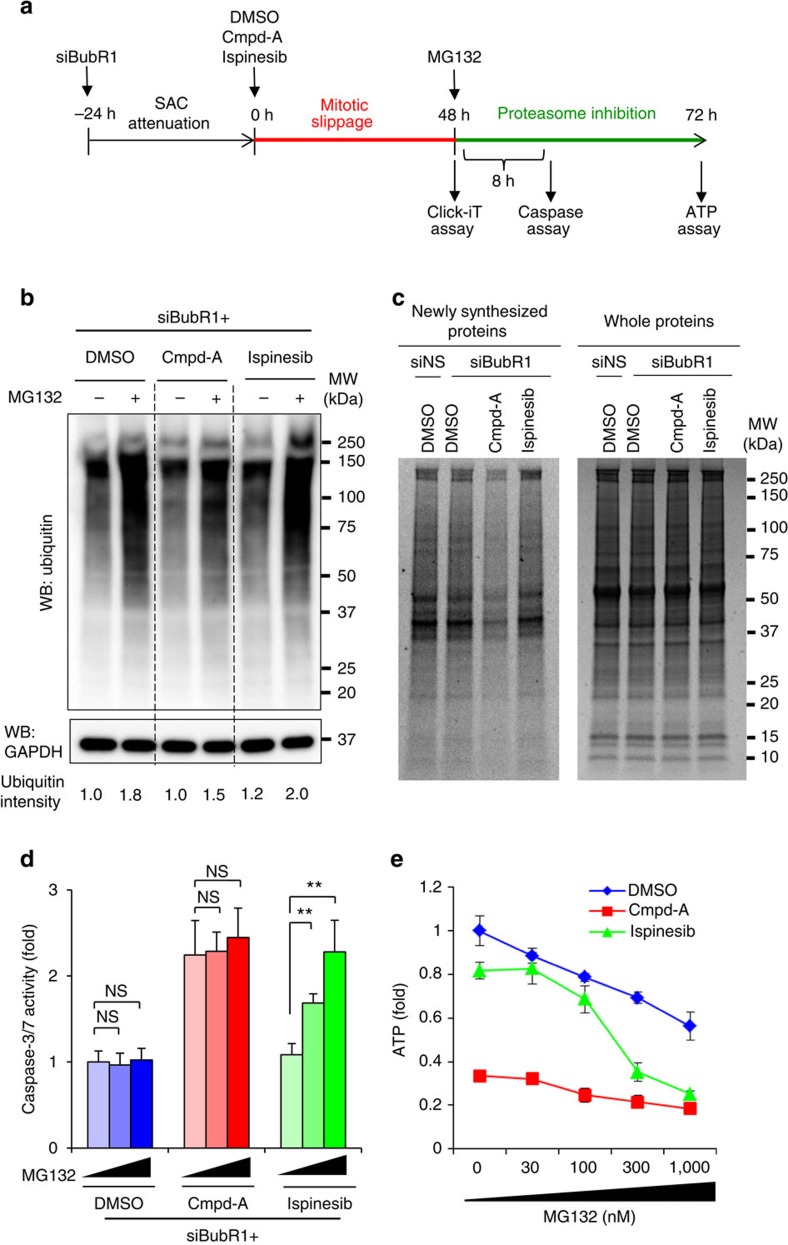
siBubR1+Cmpd-A aneuploid cells decrease global protein translation to reduce the response to the proteasome inhibitor MG132. (**a**) Schematics of the MG132 treatment experiments. (**b**) Global protein ubiquitination by MG132 in siBubR1+DMSO-, siBubR1+Cmpd-A- and siBubR1+ispinesib-treated HeLa cells. (**c**) Newly synthesized protein in siBubR1+DMSO-, siBubR1+Cmpd-A- and siBubR1+ispinesib-treated HeLa cells. Left and right gels show newly synthesized proteins detected by the Click-iT assay (Life Technologies) and all proteins detected by CBB staining as a loading control, respectively. (**d**) Effect of MG132 on caspase-3/7 activity in siBubR1+DMSO-, siBubR1+Cmpd-A- and siBubR1+ispinesib-treated HeLa cells. Blue, red and green bars indicate siBubR1+DMSO-, siBubR1+Cmpd-A- and siBubR1+ispinesib-treated HeLa cells, respectively. Relative caspase-3/7 activities were calculated in comparison with the activities in siBubR1+DMSO-treated cells. Statistical analysis was performed using Student's *t*-test. Differences were considered significant at *P*≤0.05 (*) and *P*≤0.01 (**). (**e**). Effect of MG132 on cell proliferation in siBubR1+DMSO-, siBubR1+Cmpd-A- and siBubR1+ispinesib-treated HeLa cells. Blue, red and green lines indicate siBubR1+DMSO-, siBubR1+Cmpd-A- and siBubR1+ispinesib-treated HeLa cells, respectively. Relative ATP levels were calculated in comparison with those in siBubR1+DMSO-treated cells. Relative ATP levels were calculated based on luminescence in comparison with the luminescence value for 0-nM treatment. The line plots represent mean±s.d. (*n*=3).

We also examined the effect of extraneous proteotoxic stress caused by the proteasome inhibitor MG132 on apoptosis and cell viability in siBubR1+Cmpd-A- and siBubR1+ispinesib-treated cells (Fig. 7a). Immunoblotting revealed that although MG132 treatment induced the accumulation of global ubiquitinated proteins in siBubR1+DMSO- and siBubR1+ispinesib-treated cells in a concentration-dependent manner, smaller amounts of ubiquitinated proteins were accumulated in siBubR1+Cmpd-A-treated cells (Fig. 7b). L-Azidohomoalanine incorporation assays revealed that the levels of newly synthesized proteins were considerably lower in siBubR1+Cmpd-A-treated cells than in siBubR1+DMSO- or siBubR1+ispinesib-treated cells (Fig. 7c). These data indicate that under substantial aneuploidy-associated proteotoxic stress in siBubR1+Cmpd-A-treated cells, UPR appears to downregulate the protein translation machinery to relieve this stress. As a result, these cells accumulate fewer ubiquitinated proteins in response to MG132. Accordingly, MG132 treatment neither enhanced caspase-3/7 levels nor caused growth inhibition in these aneuploid cells (Fig. 7d, red bars, and Fig. 7e, red line). Conversely, the levels of newly synthesized proteins and MG132-induced ubiquitinated proteins in siBubR1+ispinesib- and siBubR1+DMSO-treated cells were similar, although they were higher than those in siBubR1+Cmpd-A-treated cells (Fig. 7b,c). Moreover, MG132 treatment significantly elevated caspase-3/7 activity in siBubR1+ispinesib-treated cells in a concentration-dependent manner (Fig. 7d, green bars) and reduced viability to the level of siBubR1+Cmpd-A-treated cells (Fig. 7e, green and red lines). These results demonstrate that at least in these models, proteotoxic stress appears to be a key event; they also explain the different cell fates of aneuploid and polyploid cells after mitotic slippage. Although the detailed mechanisms remain unclear, polyploid cells may overdrive the protein quality control machineries to adapt to marked changes in protein homeostasis caused by the doubling of chromosome number after mitotic slippage. Such cells may be more sensitive to the modulation of protein homeostasis by proteasome inhibition.

### Cmpd-A exhibits antitumour activity in SAC-attenuated cells

**Figure 8:**
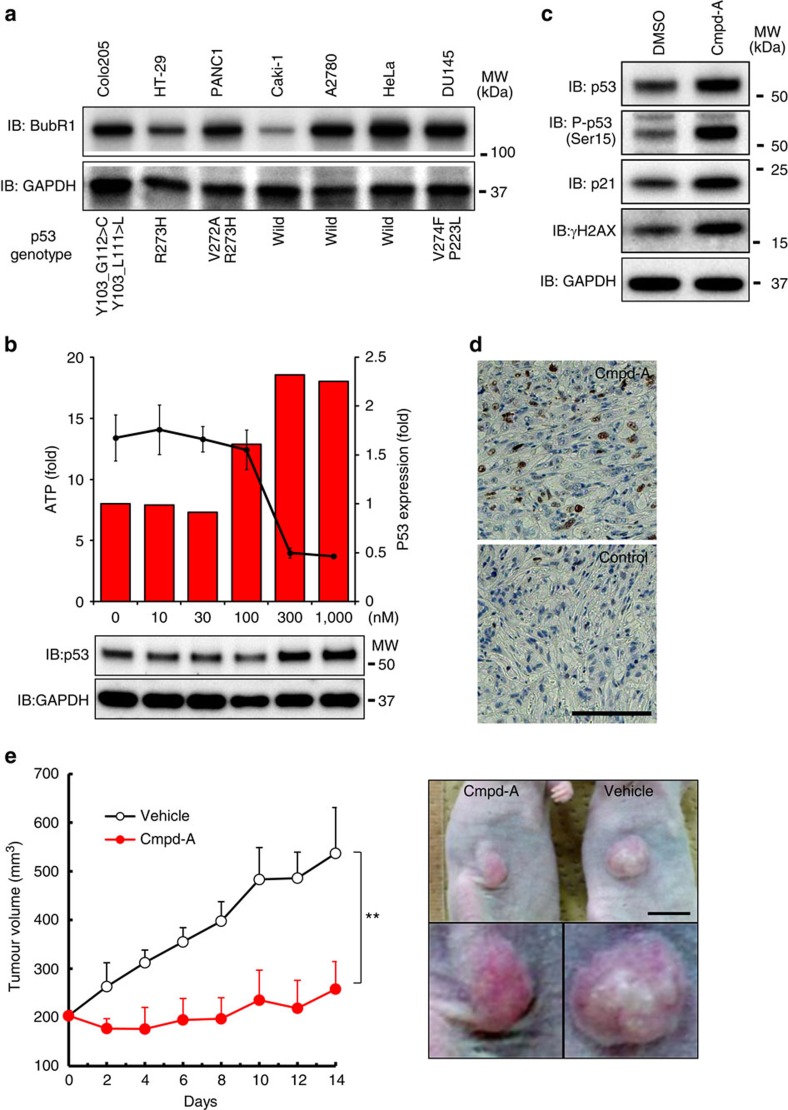
Cmpd-A increases tumour resistance in SAC-impaired Caki-1 cells *in vitro* and *in vivo*. (**a**) BubR1 downregulation in SAC-impaired Caki-1 cells. The COSMIC database was used for p53 genotyping of each cell line. (**b**) Anti-proliferative activity of Cmpd-A (black line) and p53 accumulation (red bars) in Caki-1. p53 levels were quantified using immunoblotting results. The line plots represent mean±s.d. (*n*=3). (**c**) p53 accumulation and DSBs in Caki-1 cells after Cmpd-A treatment. Cells were collected 48 h after treatment. (**d**) IHC of p53 in Caki-1 xenografts. Mice were administered 100 mg kg^−1^ Cmpd-A twice (0 and 8 h). The tumours were collected 24 h after the first administration. The white bar indicates 100 μm. (**e**) The antitumour efficacy of Cmpd-A in the Caki-1 xenograft models (right). The line plots represent mean±s.d. (*n*=5). The representative xenografts on day 8 after administration are shown (left). The black bar indicates 1 cm.

To examine spontaneous SAC attenuation, we assessed BubR1 expression in several cancer cell lines. Caki-1 cells (p53-wild-type kidney carcinoma cells) exhibited lower BubR1 expression than other cancer cell lines (Fig. 8a). FACS analysis of Caki-1 cells after Cmpd-A treatment revealed that the number of pHH3-positive cells was slightly increased 24 h after treatment (6.7% with Cmpd-A and 2.0% with DMSO). However, the numbers decreased 48 h after treatment (1.5% with Cmpd-A and 1.6% with DMSO; Supplementary Fig. 17a). These data indicate that although the SAC machinery of Caki-1 cells is transiently activated in response to CENP-E inhibition, SAC activity is eventually reduced, allowing the cells to be released from prolonged mitotic arrest induced by mitotic slippage. Similar to siBubR1-transfected HeLa cells, Caki-1 cells treated with Cmpd-A exhibited growth suppression (Fig. 8b, black line) accompanied by p53 accumulation and phosphorylation, p21 induction and increased γH2AX expression (Fig. 8b,c and Supplementary Fig. 17b). Treatment with Cmpd-A also induced time-dependent accumulation of p53, phospho-p53 and p21 in SAC-defective U87MG cells (Supplementary Fig. 17c), which exhibit less mitotic accumulation in response to colcemid (a microtubule depolymerizer). Immunostaining of the sections of a Caki-1 xenograft model revealed more p53-positive cells in Cmpd-A-treated tumours than in vehicle-treated tumours (Fig. 8d). Furthermore, Cmpd-A (at a concentration of 100 mg kg^−1^, administered three times (at 0, 8 and 24 h) on the first day of the study) displayed a potent antitumour effect (tumour growth inhibition (T/C)=16% on day 14) without significant body weight loss (Fig. 8e and Supplementary Fig. 17d). Thus, although certain cancer cells such as Caki-1 and U87MG cells spontaneously downregulate SAC proteins to attenuate the SAC machinery, CENP-E inhibition exerts anti-proliferative effects on these SAC-impaired cancer cells by activating p53 signalling pathways.

**Figure 9:**
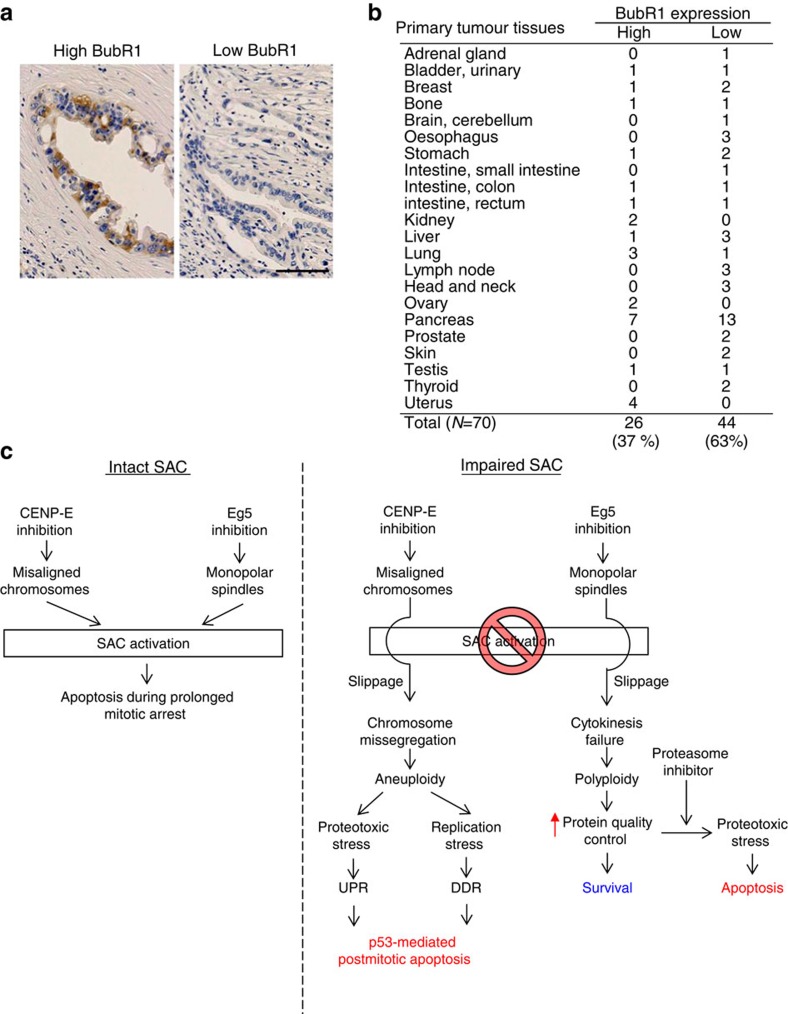
IHC of BubR1 in primary tumour tissues. (**a**) IHC of BubR1 in primary pancreatic tumours. The representative images of high (left panel) and low expression (right panel) of BubR1 are shown. The black bar indicates 100 μm. (**b**) Summary of IHC of BubR1 in 70 different tumour tissues. The tissue microarrays were purchased from BioChain Institute Inc. (**c**) Schematics of aneuploidy-mediated apoptosis after mitotic slippage.

Finally, we examined BubR1 expression in primary tumours using immunohistochemistry (IHC; Fig. 9a and Supplementary Fig. 18a). IHC revealed low BubR1 expression in 13 of the 20 primary pancreatic tumour samples and 44 of the 70 tissue samples from other tumours (Fig. 9b). These results demonstrated reduced BubR1 expression in a considerable proportion of a broad spectrum of primary tumours, although this protein was upregulated in most cancer cell lines (Fig. 8a). Furthermore, 39.4% of the primary tumours exhibited low expression of both BubR1 and p53 (Supplementary Fig. 18). Given that wild-type p53 tumours are closely associated with low p53 expression, it is possible that BubR1-depleted primary tumours carry the wild-type p53 gene. Although the SAC activity of these BubR1-depleted primary tumours remains to be elucidated, it is possible that a subset of these tumours, similar to Caki-1 cells, could attenuate the SAC machinery to escape mitotic death under mitotic inhibition.

## Discussion

In this study, we assessed the potential of CENP-E and Eg5 as cancer therapeutic targets using siRNAs and chemical inhibitors. Detailed mechanistic analyses revealed that CENP-E inhibition, but not Eg5 inhibition, induces post-mitotic effects through a unique mechanism of action involving aneuploidy-mediated p53-dependent post-mitotic apoptosis. We also demonstrated that aneuploidy generates both replication stress-mediated DSBs and proteotoxic stress to activate DDR and UPR pathways (Fig. 9c). These integrated signalling pathways, in collaboration with SAC, play important roles in eliminating chromosome instability.

Antimitotic therapies that target microtubule dynamics, such as taxanes or vinca alkaloids, are widely used in the clinical treatment of cancer^10^. Following attempts to improve the therapeutic properties of these microtubule inhibitors^46^,^47^, non-structural components of microtubules that are key components of mitosis have recently attracted attention. Several small-molecule inhibitors that induce mitotic aberrations are undergoing clinical trials^10^,^18^,^25^,^32^. Accumulated evidence suggests that the SAC machinery is responsible for the sensitivity of cancer cells to antimitotic agents^12^,^13^,^14^,^15^, although the SAC mechanism is attenuated in a broad spectrum of primary tumours (Figs 8 and 9)^48^,^49^,^50^. In this study, we demonstrated that aneuploidy caused by CENP-E inhibition has potent post-mitotic anti-proliferative p53-dependent effects on SAC-impaired cells. Polyploidy caused by Eg5 inhibition does not produce this effect. Our results are in agreement with those of some recent genetic studies reporting elevated cell death and decreased tumour formation in CENP-E^+/−^ Mad2^+/−^ double-heterozygous mice^51^. Thus, CENP-E inhibition may be effective against cancers refractory to other mitotic inhibitors, such as SAC-attenuated cancers, and it could potentially expand the therapeutic window for cancer drugs.

Although various mitotic inhibitors are being developed, the underlying mechanism of action remains unclear. First, the cellular events associated with reduced proliferation after mitotic slippage are not well understood. One hypothesis proposes that the microtubule-dependent post-mitotic checkpoint is responsible for cell cycle arrest after mitotic slippage^52^,^53^. Another controversial hypothesis proposes that prolonged mitotic arrest itself, but not the microtubule alternation checkpoint, is responsible for this phenomenon^54^,^55^. We propose an alternative hypothesis. In our model, aneuploidy-associated replication stress and proteotoxic stress also contribute to anti-proliferation in cancer cells after mitotic slippage. We revealed that siCENP-E+siBubR1 did not cause prolonged mitosis or aberrant microtubule structure formation.

Second, the mechanism by which mitotic aberration generates DNA damage is unclear. One possibility is that DNA damage accumulates during prolonged mitotic arrest^56^,^57^. However, in our model, prolonged mitotic arrest was not induced by either siBubR1+siCENP-E or siBubR1+Cmpd-A; thus, aneuploidy-induced DSBs could be generated independently of the duration of mitotic arrest. Another study proposed that the chromosome bridge during cytokinesis generates DSBs and activates DDR pathways^58^. In that study, γH2AX and 53BP1 foci were restricted to DNA positioned in or close to the cleavage furrow of cytokinesis, and most cells (∼80%) accumulated 53BP1 foci within 2 h after chromosome missegregation. In our model, DSBs were mainly generated between 24 and 48 h after mitotic slippage. In addition, larger numbers of 53BP1 foci were observed compared with the previous study (Fig. 5c). Thus, DSBs detected in our models appear to be caused by chronic chromosome instability rather than by chromosome breaks in a lagging chromosome. A third hypothesis proposes that micronuclei produced by lagging chromosomes generate DNA damage with defective and asynchronous DNA replication and aberrant DDR protein recruitment^59^. However, in our models, 53BP1 foci were mainly observed at primary nuclei rather than micronuclei (Fig. 5c). Our results demonstrate that chronic DNA replication stress after chromosome missegregation also contributes to aneuploidy-associated DSBs. In yeast, aneuploid cells display increased chromosome loss and mitotic recombination as well as defective DNA damage repair^60^. With replication stress during the S phase, the defects in DNA repair systems may increase DSB accumulation in aneuploid cells.

Third, it is unclear which molecules or pathways monitor chromosome missegregation after mitotic slippage and how these monitoring systems could exclude the aneuploid cells. Our study demonstrated that p53 activation, accompanied by aneuploidy-mediated DSBs and proteotoxic stress, is responsible for post-mitotic apoptosis excluding the aneuploid cells. Recent studies have demonstrated functional relationships between protein quality control systems and chromosome instability in aneuploid cells^38^,^39^,^61^,^62^. These results suggest that rapid changes in chromosome copy numbers could alter cellular protein homeostasis markedly. Expectedly, our model also detected phenotypes of substantial proteotoxic stress in siBubR1+Cmpd-A-treated aneuploid cells: pathological changes of ER, aggresome formation, transcriptional activation of UPR genes, spliced form of XBP1 and autophagic vesicle-associated LC3B-II (Fig. 6). Although several studies have demonstrated that ER stress could regulate p53 pathways positively or negatively, the mechanisms underlying this phenomenon remain unclear^63^,^64^,^65^,^66^. Our study revealed that p53-mediated post-mitotic apoptosis is accompanied by DDR and UPR activation (Fig. 6). UPR, in collaboration with DDR, may play an important role in eliminating aneuploid cells after mitotic slippage in a p53-dependent manner. The levels of UPR activation and autophagy were lower in polyploid cells than in aneuploid cells. Polyploid cells were more sensitive to proteasome inhibition, although the extent of ubiquitination of global proteins and the levels of new protein synthesis were similar to those in the control cells (Fig. 7). Other polyploidy-specific aspects, such as enhanced cellular metabolism^9^,^67^, may increase the sensitivity of these cells to proteotoxic stress.

In conclusion, we revealed that under SAC-defective conditions, aneuploidy caused by CENP-E inhibition, but not polyploidy induced by Eg5 inhibition, triggers p53 activation after mitotic slippage, resulting in post-mitotic suppression of proliferation. Furthermore, aneuploidy-associated DNA damage and proteotoxic stress are accompanied by p53-mediated post-mitotic apoptosis in SAC-attenuated cancer cells. Our study provides some new insights into the molecular mechanisms of cancer cell elimination by massive chromosome instability caused by mitotic inhibitors. It also demonstrates the therapeutic potential of CENP-E inhibitors as cancer drugs.

## Methods

### Compounds

Cmpd-A was synthesized by Takeda Pharmaceutical Company Ltd (Supplementary Methods). Ku55933 and ispinesib were purchased from Wako Pure Chemical Industries Ltd., and Selleck Chemicals, LLC, respectively.

### Cell growth and caspase-3/7 assays

Cell growth was evaluated by measuring protein amounts by crystal violet staining or intracellular ATP concentrations using the CellTiter-Glo luminescent cell viability assay (Promega Corp., Madison, WI, USA). Caspase-3/7 activity was evaluated using a Caspase-3/7-Glo luminescent kit (Promega). Absorbance and chemical luminescence were measured using a microplate reader.

### Immunofluorescence assay

Cells were fixed for 15 min in PBS-buffered 4% paraformaldehyde, followed by permeablization for 5 min in Triton-X buffer^68^. The following antibodies were used at a concentration of 1–2 μg ml^−1^ for immunofluorescence assays: anti-CENP-B (sc22788; Santa Cruz Biotechnology), anti-BubR1 (612503), anti-α-tubulin (T9026; Sigma-Aldrich), anti-p53 (sc126; Santa Cruz Biotechnology), anti-53BP1 (sc22760; Santa Cruz Biotechnology), anti-p62 (sc28359; Santa Cruz Biotechnology) and anti-LC3B (3868; Cell Signaling Technology). Images were captured with a Plan-APOCHROMAT × 100 oil lens under an Axiovert 200M microscope (Carl Zeiss).

### *In vivo* efficacy studies

Caki-1 cells (3–5 × 10^6^ cells per mouse) were xenografted into BALB/c nude mice (7 weeks old, female). Mice bearing tumours (100–250 mm^3^) were selected and randomly categorized into vehicle and Cmpd-A groups (5 mice per group). The xenografted mice were intraperitoneally administered 100 mg kg^−1^ Cmpd-A 3 times (at 0, 8 and 24 h) on the first day of the study. T/C (%) was calculated using the following formula:

T/C (%)=((Cmpd-A tumour volume−Cmpd-A tumour volume on day 0)/(vehicle tumour volume−vehicle tumour volume on day 0)) × 100

All *in vivo* procedures were performed in accordance with the protocols approved by the Takeda Experimental Animal Care and Use Committee.

### Time-lapse imaging and analysis

HeLa cells were cultured in 24-well culture plates with glass bottoms (MatTek Corp., Ashland, MA, USA) and transfected with siRNA oligos targeting the indicated genes. Phase-contrast images were captured every 10 min for 24 h (12–36 h after transfection). Cells were imaged using an Axiovert 200M microscope equipped with EC Plan NEOFLUAR × 20 lens (Carl Zeiss). Images were acquired and processed using AxioVision 4.5 software (Carl Zeiss). The images were converted to JPEG format and exported to Adobe Photoshop.

### FACS analysis

Cells fixed with 70% ethanol were incubated for 30 min in PBS containing 2% fetal bovine serum, 100 μg ml^−1^ RNase A (Sigma-Aldrich) and 1 μg ml^−1^ Alexa Fluor 488-conjugated pHH3 antibodies (1:50 dilution, Cat# 3465, Cell Signaling Technology, Danvers, MA, USA). After washing, the cells were incubated in 50 μg ml^−1^ propidium iodide (Sigma-Aldrich). Ten thousand cells were analysed using FACSCalibur (Becton-Dickinson, Franklin Lakes, NJ, USA).

### Detection of newly synthesized proteins and immunoblotting

Newly synthesized proteins were detected using the Click-iT reagent kit (Life Technologies) according to the manufacturer's protocol. In brief, siBubR1-transfected HeLa cells were treated with the indicated compounds for 48 h and then incubated for 30 min in methionine-free culture medium. Then, the cells were cultured for 4 h in the presence of 50 μM L-Azidohomoalanine (Life Technologies). To label the newly synthesized proteins, click reactions were performed using 25- μg aliquots of proteins from each cell lysate. Following SDS–polyacrylamide gel electrophoresis, the proteins were detected using LAS-3000 (FujiFilm). Uncropped immunoblots are shown in Supplementary Figs 19 and 20.

## Additional information

**Accession codes**: The microarray data have been deposited in the Gene Expression Omnibus under accession code GSE67905

**How to cite this article:** Ohashi, A. *et al*. Aneuploidy generates proteotoxic stress and DNA damage concurrently with p53-mediated post-mitotic apoptosis in SAC-impaired cells. *Nat. Commun.* 6:7668 doi: 10.1038/ncomms8668 (2015).

## Supplementary Material

Supplementary Information Supplementary Figures 1-20, Supplementary Tables 1-2, Supplementary Methods and Supplementary References
