# Supplementary material for: Aneuploidy generates proteotoxic stress and DNA damage concurrently with p53-mediated post-mitotic apoptosis in SAC-impaired cells
Source: Nat Commun. 2015 Jul 6;6:7668. doi: 10.1038/ncomms8668 (PMC4506520; doi:10.1038/ncomms8668)
Supplement: Supplementary Information — Supplementary Figures 1-20, Supplementary Tables 1-2, Supplementary Methods and Supplementary References [file ncomms8668-s1.pdf]

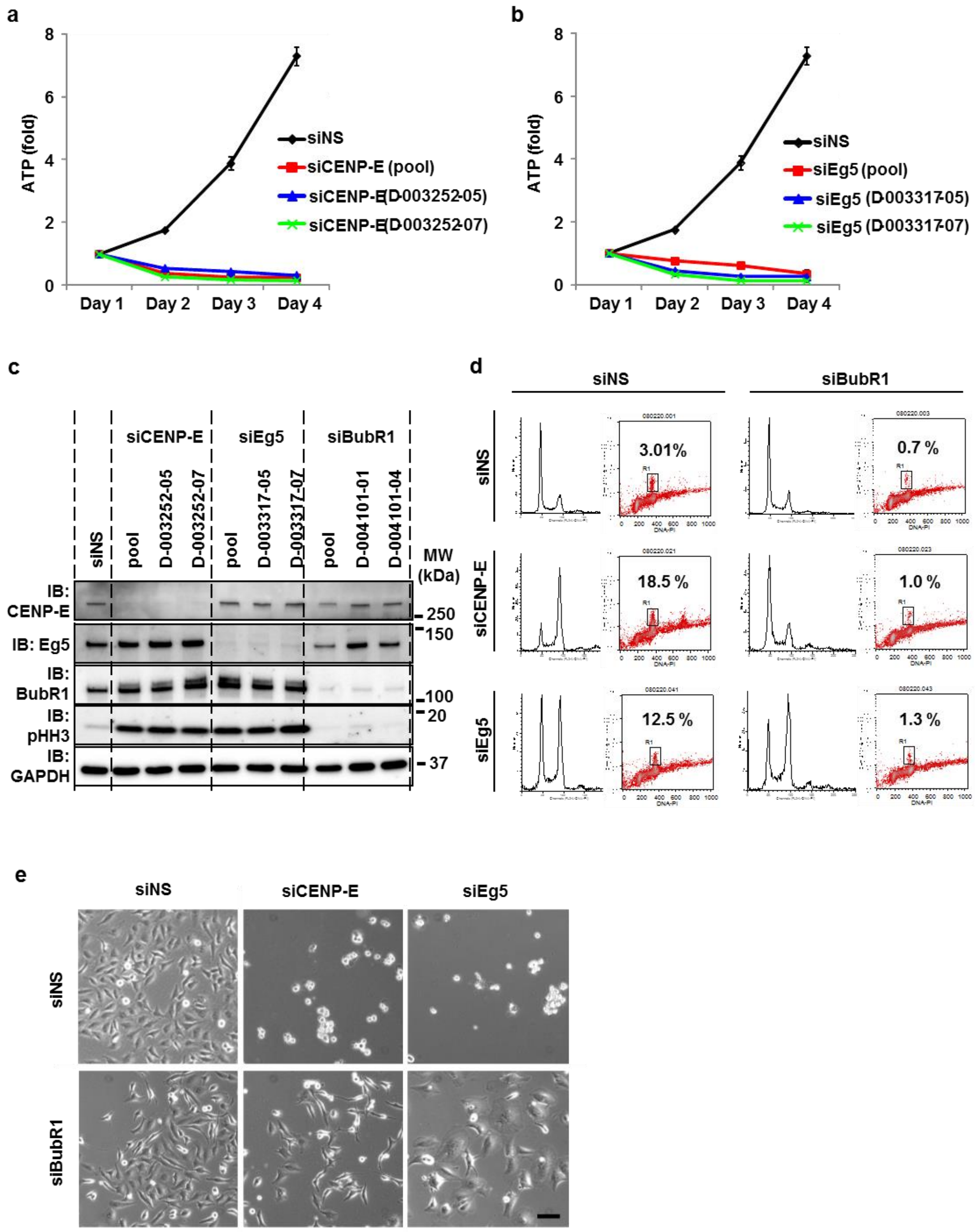
a
ATP (fold)
siNS
siCENP-E (pool)
siCENP-E (D-003252-05)
siCENP-E (D-003252-07)
Day 1
Day 2
Day 3
Day 4
b
ATP (fold)
siNS
siEg5 (pool)
siEg5 (D-003317-05)
siEg5 (D-003317-07)
c
siCENP-E
siEg5
siBubR1
siNS
pool
D-003252-05
D-003252-07
D-003317-05
D-003317-07
D-004101-01
D-004101-04
MW (kDa)
IB: CENP-E
IB: Eg5
IB: BubR1
IB: pHH3
IB: GAPDH
250
150
100
20
37
d
siNS
siBubR1
siCENP-E
siEg5
3.01%
0.7 %
18.5 %
1.0 %
12.5 %
1.3 %
e
siNS
siCENP-E
siEg5
siBubR1

Supplementary Figure 1. Knockdown of CENP-E and Eg5 caused prolonged mitotic arrest and potent growth inhibition (related to main figure 1)

a. Anti-proliferative effects in HeLa cells treated with pooled or individual siCENP-E. HeLa cells treated with pooled (pool) or individual (D-003252-05 (05), D-003252-07 (07)) siRNAs were collected on days 1-4 after the transfection. Relative ATP levels were calculated on the basis of luminescence in comparison with the day 1 luminescence values (control). The line plots represent mean ± SD (n=3). **b.** Anti-proliferative effects in HeLa cells treated with pooled or individual siEg5. HeLa cells treated with pooled (pool) or individual (D-003317-05 (05), D-003317-07 (07)) siRNAs were collected on days 1-4 after the transfection. Relative ATP levels were calculated on the basis of luminescence in comparison with the day 1 luminescence values (control). The line plots represent mean ± SD (n=3). **c.** Knockdown effect of CENP-E, Eg5 and BubR1 siRNAs. HeLa cells were transfected with the indicated siRNAs. Twenty-four hours after transfection, the cells were collected for immunoblotting. The upper form of BubR1 indicates phosphorylated BubR1. Phosphorylation of histon H3 was used as mitotic index. Immunoblotting was performed as described in Fig.1a. Immunoblotting of CENP-E and BubR1 was used to evaluate the knockdown effect of each siRNA. Immunoblotting of GAPDH was used as a loading control. **d.** Mitotic arrest of HeLa cells treated with NS, CENP-E, or Eg5 siRNAs. FACS analysis was performed 24 h after transfection. Mitotic cells were detected with antibodies against pHH3. The y-axis shows the percentage of pHH3-positive cells. **e.** Phase-contrast microscopy images of siCENP-E and siEg5 cells with or without siBubR1. Images were acquired 48 hours after e siRNA transfection. Black bar indicates 100 µm.

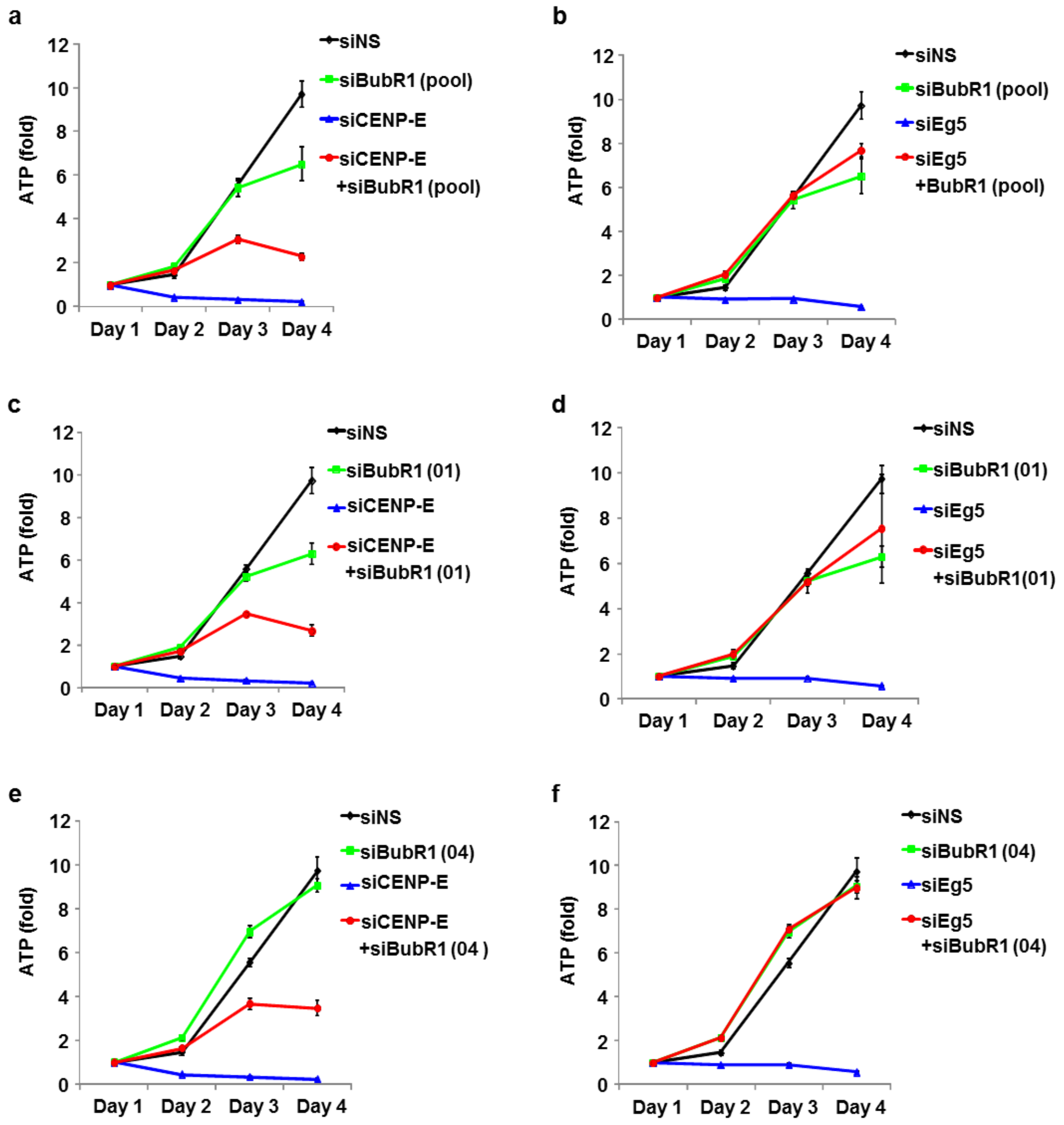
a
ATP (fold)
Day 1 Day 2 Day 3 Day 4
siNS
siBubR1 (pool)
siCENP-E
siCENP-E +siBubR1 (pool)
b
ATP (fold)
Day 1 Day 2 Day 3 Day 4
siNS
siBubR1 (pool)
siEg5
siEg5 +BubR1 (pool)
c
ATP (fold)
Day 1 Day 2 Day 3 Day 4
siNS
siBubR1 (01)
siCENP-E
siCENP-E +siBubR1 (01)
d
ATP (fold)
Day 1 Day 2 Day 3 Day 4
siNS
siBubR1 (01)
siEg5
siEg5 +siBubR1(01)
e
ATP (fold)
Day 1 Day 2 Day 3 Day 4
siNS
siBubR1 (04)
siCENP-E
siCENP-E +siBubR1 (04)
f
ATP (fold)
Day 1 Day 2 Day 3 Day 4
siNS
siBubR1 (04)
siEg5
siEg5 +siBubR1 (04)

Supplementary Figure 2. Anti-proliferative effects of pooled or individual siRNAs of BubR1 in the CENP-E- or Eg5-knockdown HeLa cells. (related to main figure 1)

a-f. Pooled siCENP-E (a, c and e) and siEg5 (b, d and f) were transfected into HeLa cells in combination with pooled (pool; a-b) or individual (D-004101-01 (01); c-d, D-004101-04 (04); e-f) siBubR1. The cells transfected with the indicated siRNAs were collected on days 1-4 after transfection. The cell viability was evaluated based on the intracellular ATP concentrations using the CellTiter-Glo luminescent cell-viability assay kit (Promega). The chemical luminescence was measured with a microplate reader. The y-axis indicates Relative ATP levels were, calculated on the basis of luminescence in comparison with the day 1 luminescence values (control). The line plots represent mean ± SD (n=3).

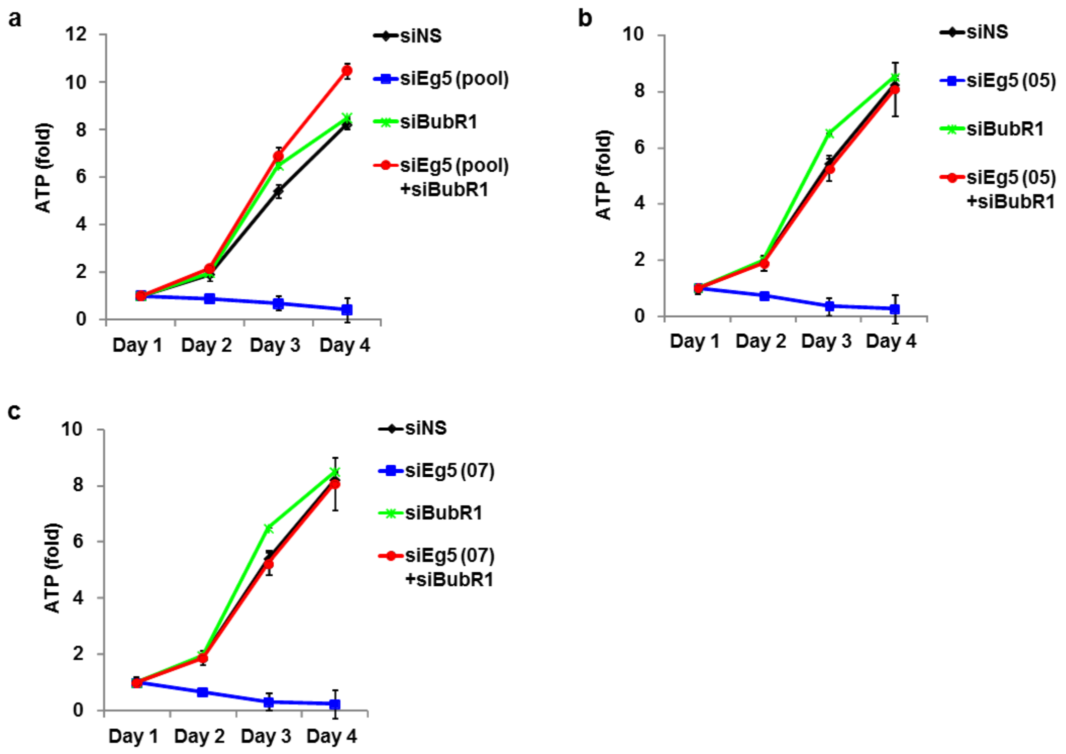
a
ATP (fold)
12
10
8
6
4
2
0
Day 1
Day 2
Day 3
Day 4
siNS
siEg5 (pool)
siBubR1
siEg5 (pool) +siBubR1
b
ATP (fold)
10
8
6
4
2
0
Day 1
Day 2
Day 3
Day 4
siNS
siEg5 (05)
siBubR1
siEg5 (05) +siBubR1
c
ATP (fold)
10
8
6
4
2
0
Day 1
Day 2
Day 3
Day 4
siNS
siEg5 (07)
siBubR1
siEg5 (07) +siBubR1

Supplementary Figure 3. Anti-proliferative effects of siEg5 in the BubR1-knockdown HeLa cells. (related to main figure 1)

a-c. Pooled (a) or individual (D-003317-05 (b) and D-003317-07 (c)) siEg5 were transfected into HeLa cells in combination with pooled siBubR1. The cells transfected with the indicated siRNAs were collected on days 1-4 after transfection. The cell viability was evaluated based on the intracellular ATP concentrations using the CellTiter-Glo luminescent cell-viability assay kit (Promega). The chemical luminescence was measured with a microplate reader. The y-axis indicates relative ATP levels, calculated on the basis of luminescence in comparison with the day 1 luminescence values (control). The line plots represent mean ± SD (n=3).

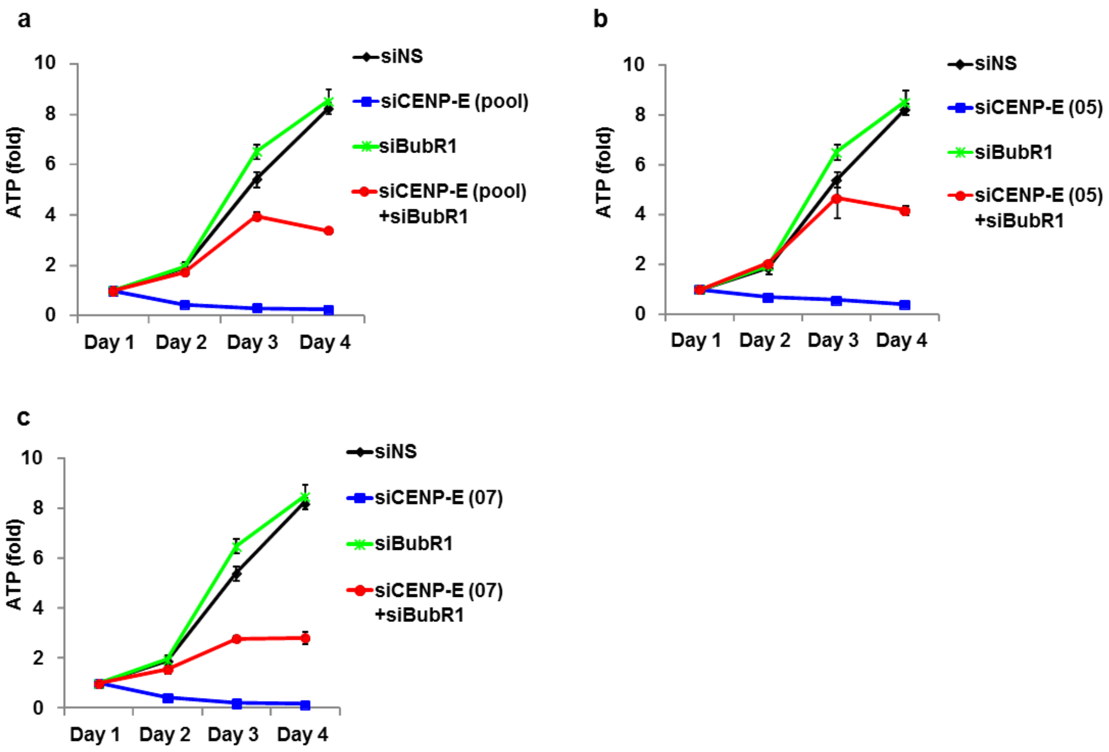
a
ATP (fold)
10
8
6
4
2
0
Day 1
Day 2
Day 3
Day 4
siNS
siCENP-E (pool)
siBubR1
siCENP-E (pool)
+siBubR1
b
ATP (fold)
10
8
6
4
2
0
Day 1
Day 2
Day 3
Day 4
siNS
siCENP-E (05)
siBubR1
siCENP-E (05)
+siBubR1
c
ATP (fold)
10
8
6
4
2
0
Day 1
Day 2
Day 3
Day 4
siNS
siCENP-E (07)
siBubR1
siCENP-E (07)
+siBubR1

Supplementary Figure 4. Anti-proliferative effects of pooled or individual siRNAs of CENP-E in the BubR1-knockdown HeLa cells. (related to main figure 1)

a-c. Pooled (a) or individual (D-003252-05 (b) and D-003252-07 (c)) siCENP-E were transfected into HeLa cells in combination with pooled siBubR1. The cells transfected with the indicated siRNAs were collected on days 1-4 after transfection. The cell viability was evaluated based on the intracellular ATP concentrations using the CellTiter-Glo luminescent cell-viability assay kit (Promega). The chemical luminescence was measured with a microplate reader. The y-axis indicates relative ATP levels, calculated on the basis of luminescence in comparison with the day 1 luminescence values (control). The line plots represent mean ± SD (n=3).

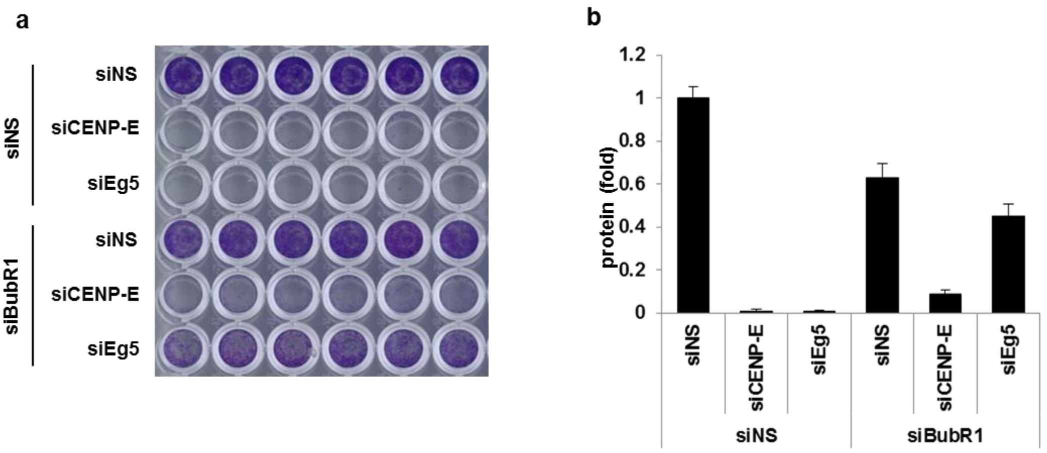
a
siNS
siBubR1
siNS
siCENP-E
siEg5
siNS
siCENP-E
siEg5
b
protein (fold)
1.2
1
0.8
0.6
0.4
0.2
0
siNS
siCENP-E
siEg5
siNS
siCENP-E
siEg5
siNS
siBubR1

Supplementary Figure 5. Crystal violet staining in siCENP-E and siEg5 cells with or without siBubR1. (related to main figure 1)

a. Images of crystal violet staining in HeLa cells transfected with the indicated siRNAs. Cells were collected on day 5 after transfection for crystal violet staining. **b.** Quantified data form Supplementary Fig. 5a. The absorbance of crystal violet was measured with a microplate reader. The y-axis indicates the normalized protein amounts, calculated on the basis of absorbance in comparison with the siNS absorbance value (control). The data represent mean ± SD (n=6).

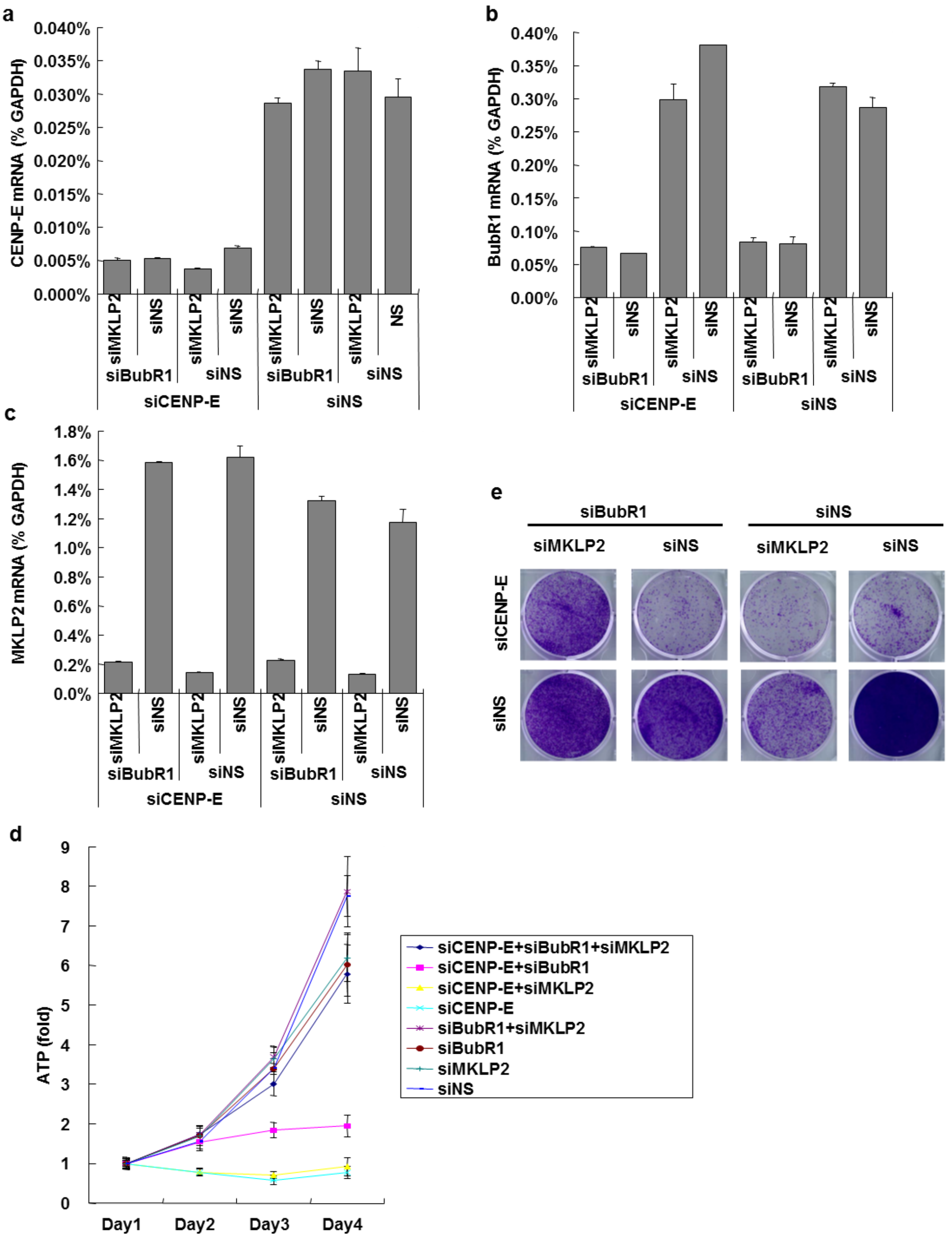

a
CENP-E mRNA (% GAPDH)
0.040%
0.035%
0.030%
0.025%
0.020%
0.015%
0.010%
0.005%
0.000%
siMKLP2
siNS
siMKLP2
siNS
siMKLP2
siNS
siMKLP2
NS
siBubR1
siNS
siBubR1
siNS
siCENP-E
siNS
b
BubR1 mRNA (% GAPDH)
0.40%
0.35%
0.30%
0.25%
0.20%
0.15%
0.10%
0.05%
0.00%
siMKLP2
siNS
siMKLP2
siNS
siMKLP2
siNS
siMKLP2
siNS
siBubR1
siNS
siBubR1
siNS
siCENP-E
siNS
c
MKLP2 mRNA (% GAPDH)
1.8%
1.6%
1.4%
1.2%
1.0%
0.8%
0.6%
0.4%
0.2%
0.0%
siMKLP2
siNS
siMKLP2
siNS
siMKLP2
siNS
siMKLP2
siNS
siBubR1
siNS
siBubR1
siNS
siCENP-E
siNS
e
siBubR1
siNS
siMKLP2
siNS
siMKLP2
siNS
siCENP-E
siNS
d
ATP (fold)
9
8
7
6
5
4
3
2
1
0
Day1
Day2
Day3
Day4
siCENP-E+siBubR1+siMKLP2
siCENP-E+siBubR1
siCENP-E+siMKLP2
siCENP-E
siBubR1+siMKLP2
siBubR1
siMKLP2
siNS

Supplementary Figure 6. Knockdown efficiency of CENP-E, BubR1, and MKLP2 siRNAs (related to main figure 2)

a-c. HeLa cells were co-transfected with CENP-E, BubR1, MKLP2, and NS siRNAs. Forty-eight hours after transfection, the cells were collected. TaqMan RT-PCR of CENP-E (a), BubR1 (b), and MKLP2 (c) was performed using an ABI PRISM 7000 instrument (Applied Biosystems). The expression ratios were quantified by dividing the expression level of the indicated genes by the expression level of GAPDH. The data represent mean ± SD (n=3). **d.** HeLa cells transfected with CENP-E, BubR1, and MKLP2 siRNAs (dark blue); CENP-E and BubR1 siRNAs (pink); CENP-E and MLKP2 siRNAs (yellow); CENP-E siRNA (light blue); BubR1 and MKLP2 siRNAs (purple); BubR1 (brown); MKLP2 (green); or NS siRNA (blue) were collected on days 1-4 after transfection. The cell growth was evaluated based on the intracellular ATP concentrations using the Cell Titer-Glo luminescent cell viability assay kit (Promega). The chemical luminescence was measured with a microplate reader. The y-axis indicates relative ATP levels, calculated as the luminescence, in comparison with the day 1 luminescence value (control). The line plots represent mean ± SD (n=3). **e.** Picture of crystal violet staining in HeLa cells with the indicated siRNAs. Cells transfected with the indicated siRNA were collected on days 7 after transfection.

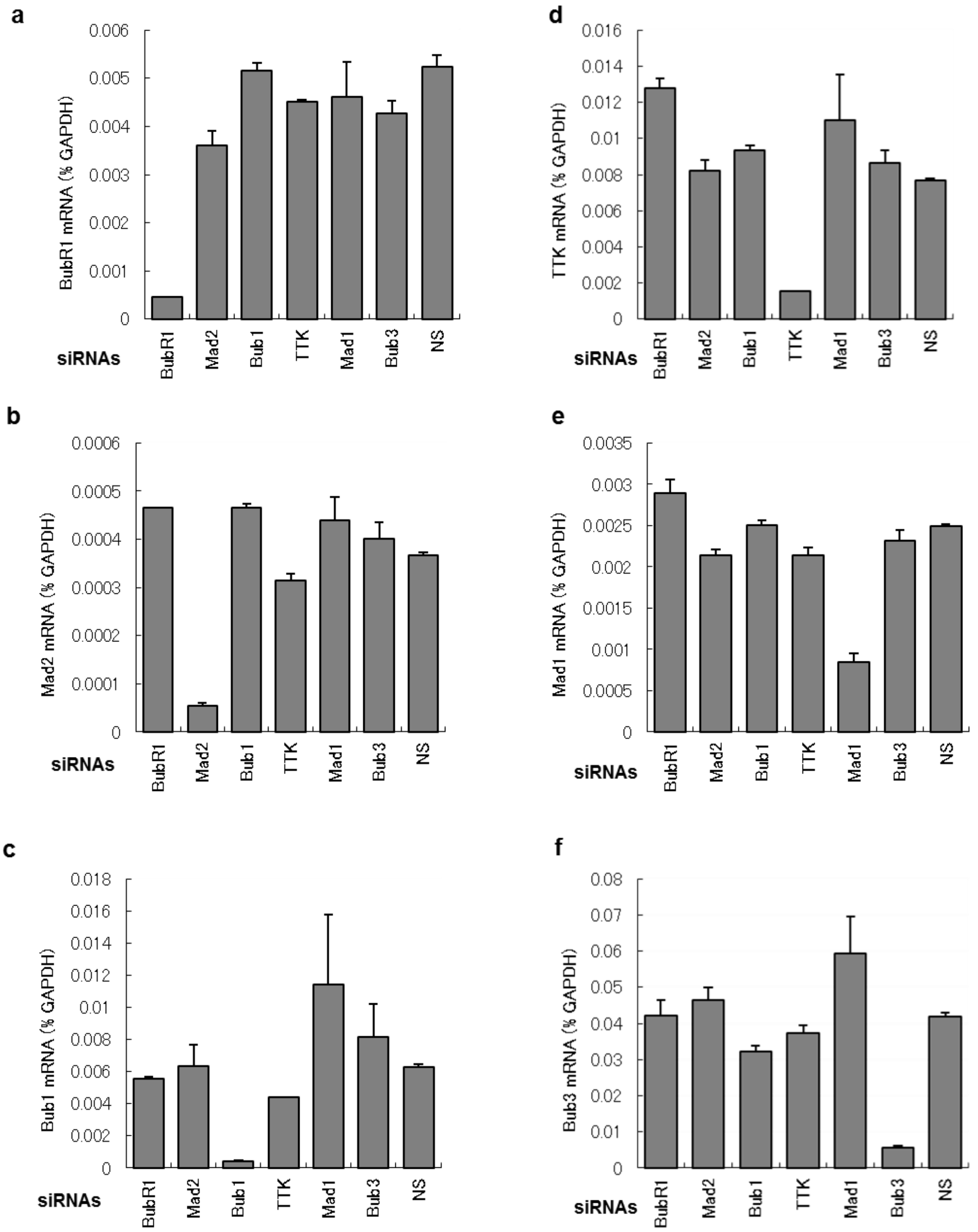
a
BubR1 mRNA (% GAPDH)
0.006
0.005
0.004
0.003
0.002
0.001
0
siRNAs
BubR1
Mad2
Bub1
TTK
Mad1
Bub3
NS
b
Mad2 mRNA (% GAPDH)
0.0006
0.0005
0.0004
0.0003
0.0002
0.0001
0
c
Bub1 mRNA (% GAPDH)
0.018
0.016
0.014
0.012
0.01
0.008
0.006
0.004
0.002
d
TTK mRNA (% GAPDH)
0.016
0.014
0.012
0.01
0.008
0.006
0.004
0.002
e
Mad1 mRNA (% GAPDH)
0.0035
0.003
0.0025
0.002
0.0015
0.001
0.0005
f
Bub3 mRNA (% GAPDH)
0.08
0.07
0.06
0.05
0.04
0.03
0.02
0.01

Supplementary Figure 7. Knockdown efficiency of BubR1, Mad2, Bub1, TTK, Mad1 and Bub3 siRNAs (related to main figure 2)

a-f. HeLa cells were transfected with BubR1, Mad2, Bub1, TTK, Mad1, Bub3, and NS siRNAs. Forty-eight hours after transfection, the cells were collected. TaqMan RT-PCR for BubR1 (a), Mad2 (b), Bub1 (c), TTK (d), Mad1 (e), and Bub3 (f) was performed using an ABI PRISM 7000 instrument (Applied Biosystems). The expression ratios were quantified by dividing the expression level of the indicated genes by the expression level of GAPDH. The line plots represent mean ± SD (n=3).

a

siBubR1	+	+	+
siCENP-E	-	+	-
siEg5	-	-	+
Up-regulated	51	434	149
Down-regulated	7	317	143

b

Number of transcripts regulated by p53 (probe number)

siBubR1	+	+	+
siCENP-E	-	+	-
siEg5	-	-	+
Up-regulated	13	52	27
Down-regulated	0	4	2

c

Genes	Fold
GADD45A	4.59
P21	3.45
Cyclin D1	2.80
TIMP	1.19
BAX	1.12
CDK4	-1.01
P53	-1.10
CDK2	-1.13
PCNA	-1.17
RB1	-1.26
Cyclin E1	-1.29
ATM	-1.42
APAF1	-1.46

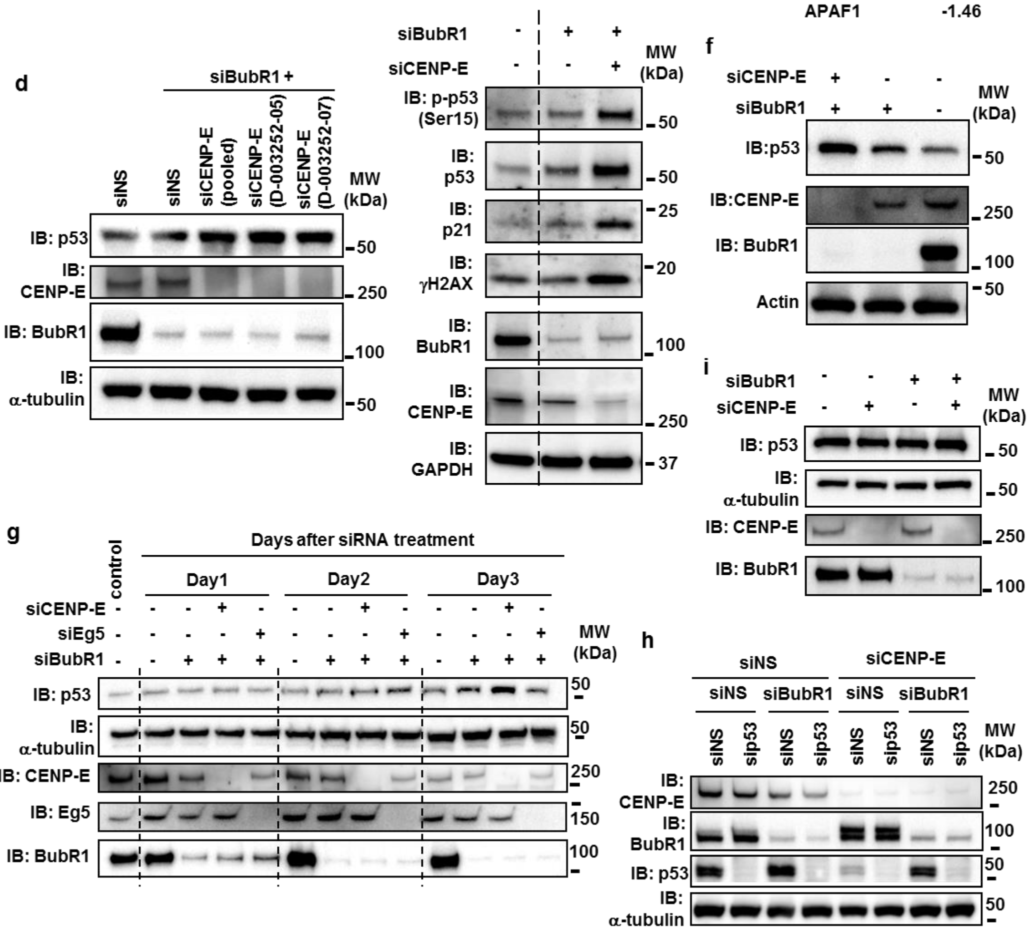

Supplementary Figure 8. Comprehensive gene expression analysis (related to main figure 3)

a. Overview of the modification of gene expression in siBubR1-transfected cells, siCENP-E+siBubR1-transfected cells, and siEg5+siBubR1-transfected cells. Upregulated and downregulated indicate >2-fold increased and >2-fold decreased expression of transcript compared with siNS-transfected cells. **b.** Overview of the modification of p53-regulatory gene expression. The fold change was quantified as described in Supplementary Fig 8a. **c.** Fold changes of p53-regulatory gene expression in siCENP-E+siBubR1-transfected cells compared with siNS-transfected cells. **d.** Protein expression of p53 in siCENP-E+siBubR1-transfected cells. HeLa cells treated with pooled (pool) or individual (D-003252-05, D-003252-07) siCENP-E with pooled siBubR1 were collected on 72 hours after the siRNA transfection for immunoblotting. Immunoblotting of CENP-E and BubR1 was used to evaluate the knockdown effect of each siRNA. Immunoblotting of α-Tubulin was used as a loading control. **e.** Protein expression of p53 in siCENP-E+siBubR1-transfected H460 cells. H460 cells were treated with siCENP-E, siBubR1 or siNS (-). Seventy-two hours after the siRNA transfection, the cells were collected for immunoblotting. Immunoblotting of CENP-E and BubR1 was used to evaluate the knockdown effect of each siRNA. Immunoblotting

of GAPDH was used as a loading control. **f.** Protein expression of p53 in siCENP-E+siBubR1 HCT116 cells. HCT116 cells were treated with siCENP-E, siBubR1 or siNS (-). Seventy-two hours after the siRNA transfection, the cells were collected for immunoblotting. Immunoblotting of CENP-E and BubR1 was used to evaluate the knockdown effect of each siRNA. Immunoblotting of GAPDH was used as a loading control. **g.** Time-dependent change of p53 expression in siCENP-E+siBubR1 cells. Cells transfected with the indicated siRNAs were collected on day 1, day 2, and day 3 after transfection. Immunoblotting of p53, CENP-E, Eg5, BubR1, and α-tubulin was performed. CENP-E, Eg5, and BubR1 were used to evaluate the knockdown effect of each siRNA. Immunoblotting of α-Tubulin was used as a loading control. **h.** Cells transfected with the indicated siRNAs were collected 48 h after transfection. CENP-E, BubR1 and p53 were used to evaluate the knockdown effect of each siRNA. α-Tubulin was used as a loading control. **i.** p53 expression in siCENP-E+siBubR1 SKBR3. Cells for immunoblotting were collected on day 3 after transfection.

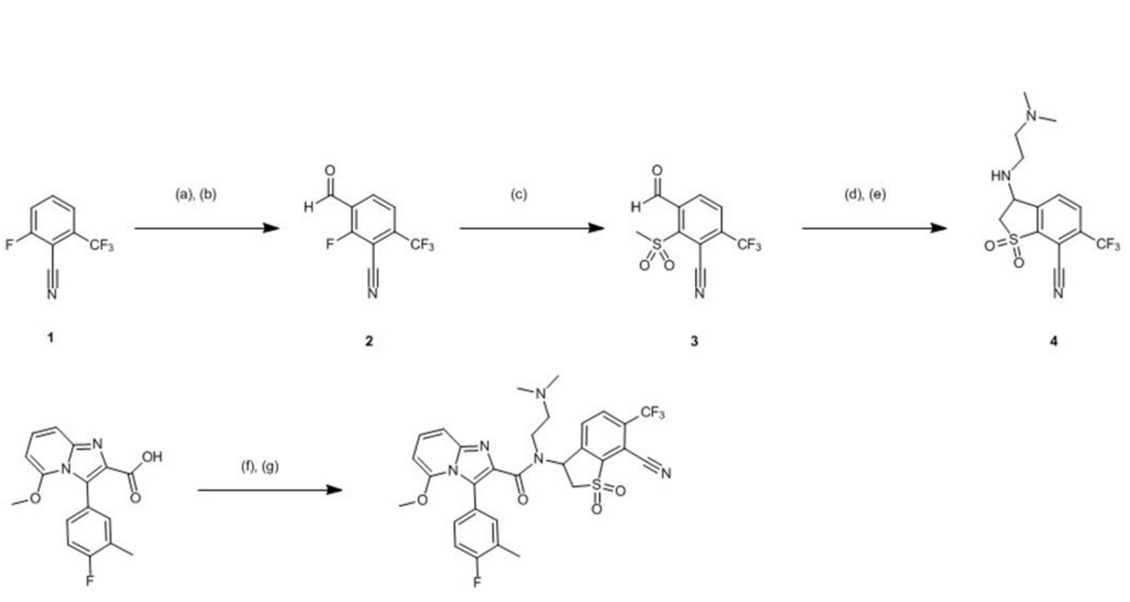

Compound-A

(+)-enantiomer

Supplementary Figure 9. Preparation of Compound-A (related to main figure 4)

Reagents and conditions are as follows: (a) *n*-BuLi/hexanes, 2,2,6,6-tetramethylpiperidine, THF, –50 ºC, 30 min, then **1**, THF, –50 ºC, 30 min; (b) *N*,*N*-dimethylformamide, THF, –50 ºC, 15 min, then –10 ºC, 20 min; (c) **2**, sodium methanesulphinate, DMSO, room temp., 2.5 h; (d) **3**, *N*,*N*-dimethylethane-1,2-diamine, THF, room temp., 2 h; (e) LDA/hexanes/THF, 0 ºC, 50 min; (f) **4**, **5**, HATU, DIEA, DMF, 50 ºC, 6 h; (g) preparative SFC (CHIRALCEL ODH (KC003)).

a

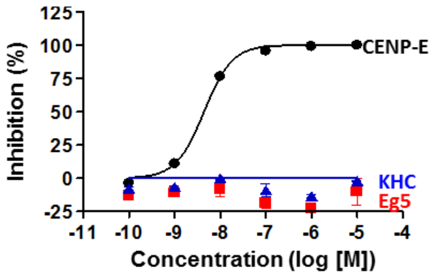

b

Kinases	Inhibition at 1000 nM (%)	IC_{50} Values
AKT1	-7	> 1000 nM
ASK1	-10	> 1000 nM
Aurora-B	-1	> 1000 nM
BRAF	-2	> 1000 nM
CDC7	1	> 1000 nM
CDK1	-9	> 1000 nM
CDK2	3	> 1000 nM
CHK1	3	> 1000 nM
CK1δ	2	> 1000 nM
c-Kit	-8	> 1000 nM
c-Met	-5	> 1000 nM
CSK	-4	> 1000 nM
EGFR	-8	> 1000 nM
EPHA5	-3	> 1000 nM
ERK1	-12	> 1000 nM
FAK	-5	> 1000 nM
FGFR1	-4	> 1000 nM
GSK3β	5	> 1000 nM
IKKβ	9	> 1000 nM
INSR	-3	> 1000 nM
JAK1	-3	> 1000 nM
JNK1	8	> 1000 nM
MAPKAPK2	16	> 1000 nM
MEK1	4	> 1000 nM
MEKK1	-19	> 1000 nM
NEK2	2	> 1000 nM
p38α	-3	> 1000 nM
PDGFRβ	-5	> 1000 nM
PI3Kα	0	> 1000 nM
PKA	6	> 1000 nM
PLK1	-1	> 1000 nM
ROCK1	-1	> 1000 nM
SRC	-3	> 1000 nM
SRPK1	-9	> 1000 nM
Tie2	-2	> 1000 nM
VEGFR2	-3	> 1000 nM

Supplementary Figure 10. Enzymatic and Cellular profiles of CENP-E selective inhibitor Compound-A (related to main figure 4)

a. Compound-A inhibition of CENP-E (black), Eg5 (red), and kinesin heavy chain (KHC) (blue). The line plots represent mean ± SD (n=3). **b.** Enzymatic selectivity profiles of Compound-A on selected kinases. The enzyme assays were performed using 36 kinases. Compound-A at 1000 nM did not inhibit ATPase activities of all 36 kinases.

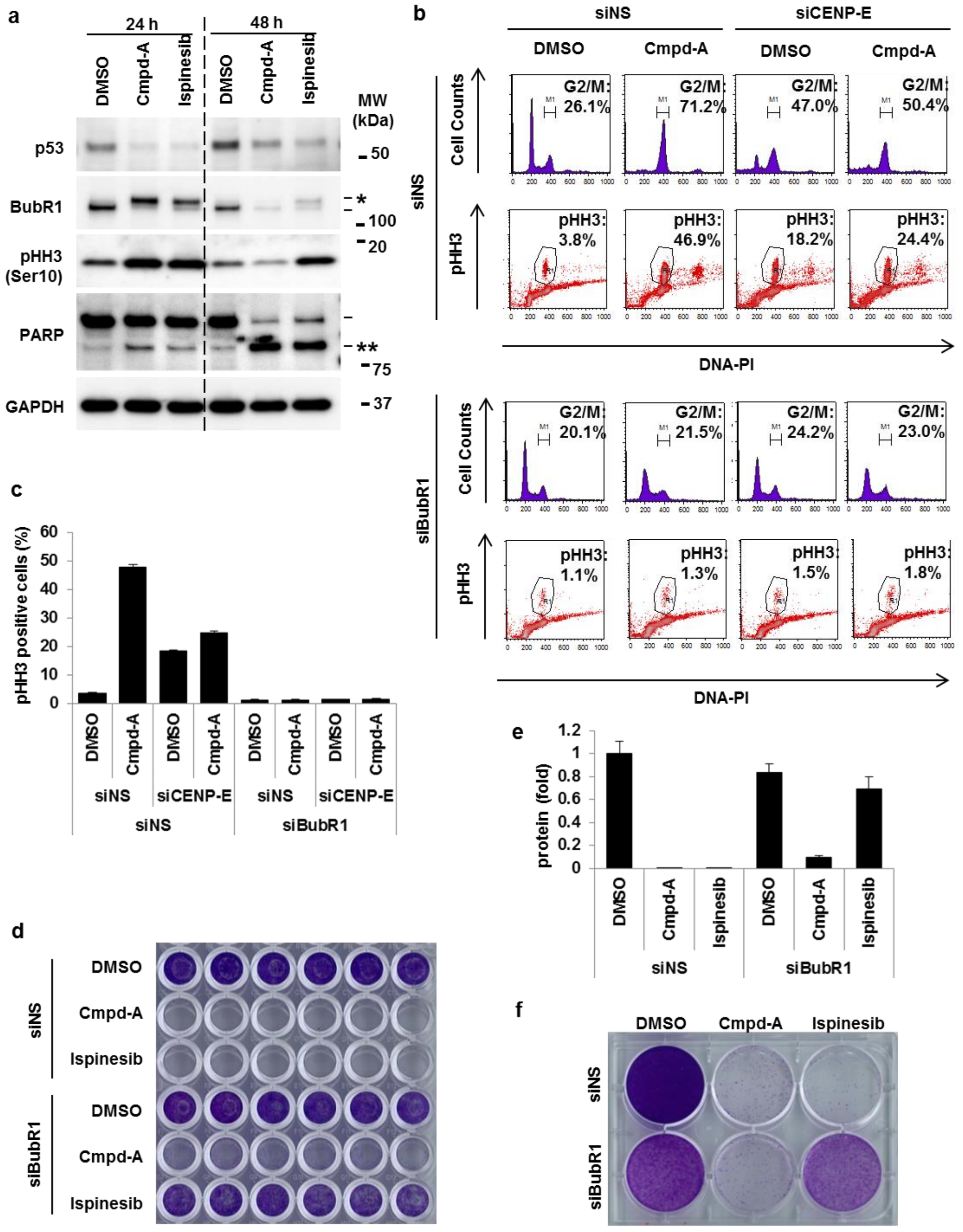
a
24 h
48 h
DMSO
Cmpd-A
Ispinesib
MW (kDa)
p53
BubR1
pHH3 (Ser10)
PARP
GAPDH
50
*
100
20
**
75
37
b
siNS
siCENP-E
DMSO
Cmpd-A
Cell Counts
pHH3
G2/M: 26.1%
G2/M: 71.2%
G2/M: 47.0%
G2/M: 50.4%
pHH3: 3.8%
pHH3: 46.9%
pHH3: 18.2%
pHH3: 24.4%
DNA-PI
siBubR1
G2/M: 20.1%
G2/M: 21.5%
G2/M: 24.2%
G2/M: 23.0%
pHH3: 1.1%
pHH3: 1.3%
pHH3: 1.5%
pHH3: 1.8%
c
pHH3 positive cells (%)
60
50
40
30
20
10
0
e
protein (fold)
1.2
1
0.8
0.6
0.4
0.2
0
d
f

Supplementary Figure 11. Effects of pharmacological inhibition of CENP-E and Eg5 on mitotic arrest and anti-proliferation in HeLa cells (related to main figure 4)

a. HeLa cells were treated with Cmpd-A (200 nM) or Ispinesib (10 nM) for 24 and 48 hours. * and ** indicate phosphorylated BubR1 and cleaved PARP, respectively. **b.** Effects of co-treatment with Cmpd-A and siCENP-E on mitotic arrest in Hela cells. Twenty-four hours after siRNA transfection, the cells were treated with or without Cmpd-A (200 nM) for 24 hours. **c.** Quantification of pHH3 positive cells in Supplementary Fig. 11b. The data was quantified with the triplicated experiments. The data represent mean ± SD (n=3). **d.** Images of crystal violet staining in HeLa cells treated with Cmpd-A or Ispinesib. Twenty-four hours after siRNA transfection, the cells were treated with Cmpd-A (200 nM) or Ispinesib (10 nM) for 120 hours. **e.** Quantified data form Supplementary Fig. 11d. The absorbance of crystal violet was measured with a microplate reader. The y-axis indicates the normalized protein amounts, calculated as absorbance, in comparison with the siNS+DMSO absorbance value (control). The line plots represent mean ± SD (n=6). **f.** Pictures of crystal violet staining in Cmpd-A and Ispinesib-treated HeLa cells with or without siBubR1. Twenty-four hours after siRNA transfection, the cells were treated with Cmpd-A (200 nM) or Ispinesib (10 nM) for 7 days

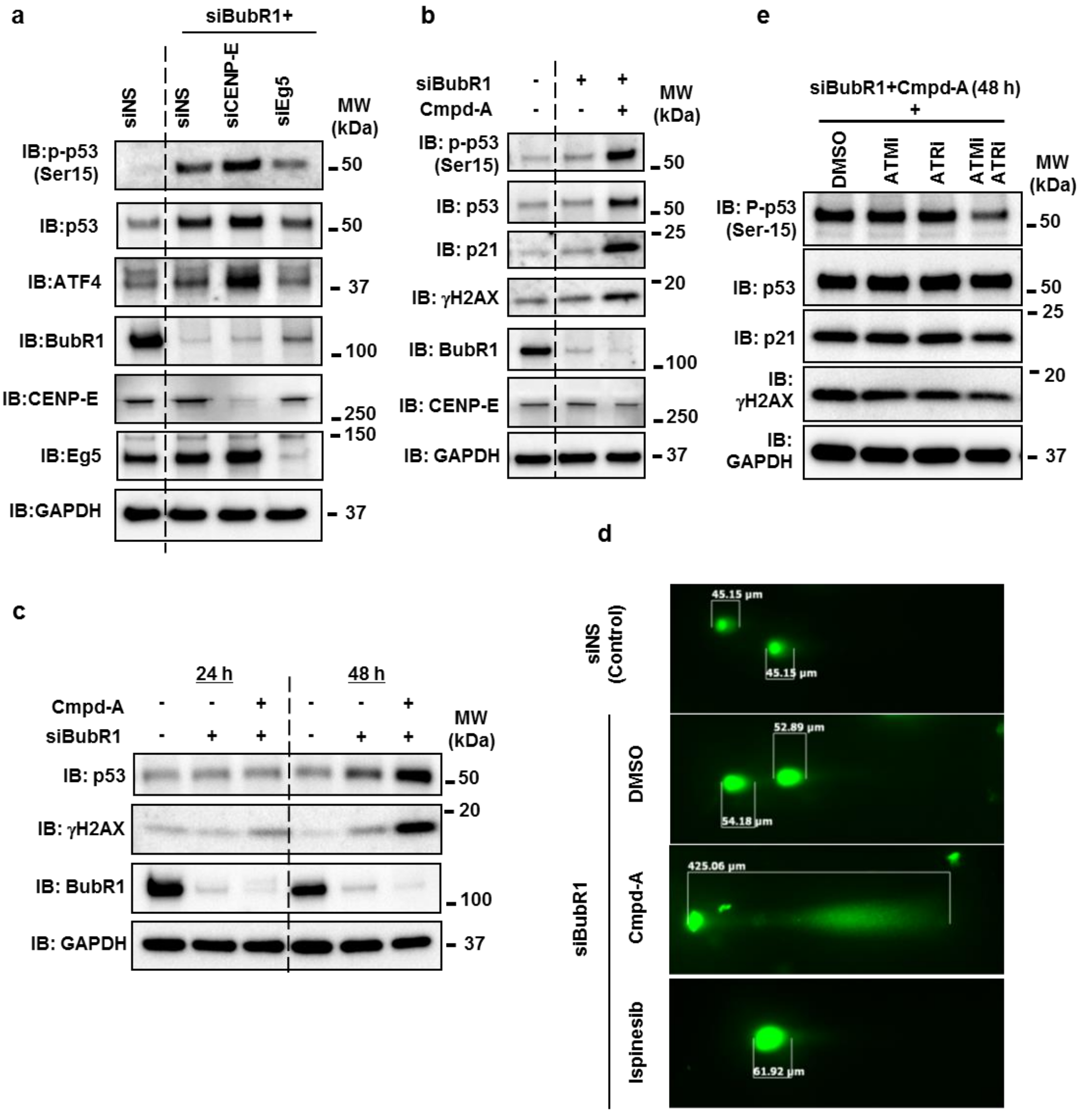
a
siBubR1+
siNS
siNS
siCENP-E
siEg5
MW (kDa)
IB:p-p53 (Ser15)
IB:p53
IB:ATF4
IB:BubR1
IB:CENP-E
IB:Eg5
IB:GAPDH
50
50
37
100
250
150
37
b
siBubR1
Cmpd-A
MW (kDa)
IB: p-p53 (Ser15)
IB: p53
IB: p21
IB: γH2AX
IB: BubR1
IB: CENP-E
IB: GAPDH
50
50
25
20
100
250
37
e
siBubR1+Cmpd-A (48 h)
+
DMSO
ATMi
ATRi
ATMi ATRi
MW (kDa)
IB: P-p53 (Ser-15)
IB: p53
IB: p21
IB: γH2AX
IB: GAPDH
50
50
25
20
37
c
24 h
48 h
Cmpd-A
siBubR1
MW (kDa)
IB: p53
IB: γH2AX
IB: BubR1
IB: GAPDH
50
20
100
37
d
siNS (Control)
45.15 μm
45.15 μm
siBubR1
DMSO
52.89 μm
54.18 μm
Cmpd-A
425.06 μm
Ispinesib
61.92 μm

Supplementary Figure 12. DNA damage accumulation after Compound-A treatment in the BubR1-knockdown cells (related to main figure 5)

a. Protein expression of p53, phosphorylated p53, and ATF4 in siCENP-E+ siBubR1-transfected HeLa cells. Cells for immunoblotting were collected on day 3 after transfection. **b.** Accumulation of p53 protein by Compound-A treatment in siBubR1-transfected H460 cells. Twenty-four hours after siRNA transfection, the cells were treated with or without Cmpd-A (200 nM) for 48 hours. **c.** Timing of p53 induction by Compound-A treatment in siBubR1-transfected HeLa cells. Cells were collected 24 or 48 h after Compound-A treatment. **d.** Representative images of neutral comet assay in siBubR1+Cmpd-A-treated cells. Twenty-four hours after siRNA transfection, the cells were treated with DMSO, Cmpd-A (200 nM), or Ispinesib (10 nM) for 72 hours. The length of DNA tails was measured by AxioVision. **e.** Phosphorylation of p53 by ATM and ATR. The cells were treated with ATM and ATR inhibitors for 1 h following 48 h of treatment with Cmpd-A.

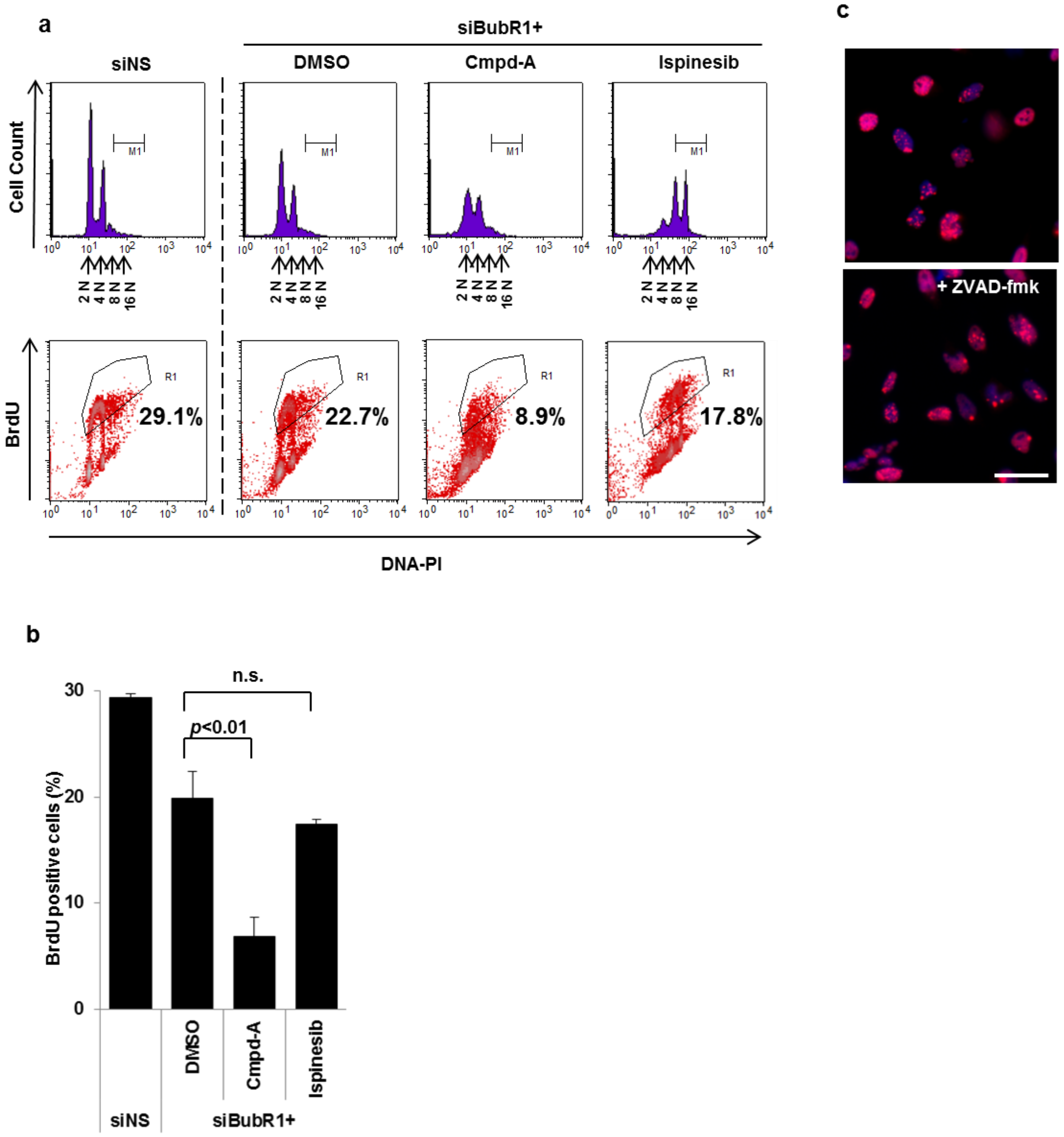
a
siBubR1+
siNS
DMSO
Cmpd-A
Ispinesib
Cell Count
M1
2 N
4 N
8 N
16 N
BrdU
R1
29.1%
22.7%
8.9%
17.8%
DNA-PI
b
n.s.
p<0.01
BrdU positive cells (%)
30
20
10
0
DMSO
Cmpd-A
Ispinesib
siNS
siBubR1+
c
+ ZVAD-fmk

Supplementary Figure 13. BrdU incorporation in siBubR1+Cmpd-A or siBubR1+Eg5 cells (related to main figure 5)

a. Cell cycle analysis in siBubR1+Cmpd-A- or siBubR1+Ispinesib-treated cells. Twenty-four hours after siRNA transfection, the cells were treated with DMSO, Cmpd-A (200 nM), or Ispinesib (10 nM) for 72 hours. BrdU was incorporated into the drug-treated cells for 15 min, and then the cells were collected for FACS analysis. The X-axis indicate log-scaled DNA-PI. **b.** The BrdU positive cells (R1) in Supplementary Fig. 13a were quantified. The data represent mean ± SD (n=3). Statistical analysis was performed using Student's t-test. Differences were considered significant at $p \leq 0.05$ (*) and $p \leq 0.01$ (**). **c.** Immunofluorescence of 53BP1 in siBubR1+Cmpd-A HeLa cells with or without Caspase inhibitor ZVAD-fmk. The siBubR1-transfected cells were treated with 200 nM Compound-A in presence (upper) or absence (lower) of 2 μM of ZVAD-fmk for 48 h. Immunofluorescence measurements of 53BP1 were performed as described in Fig. 1f. White bar indicates 50 μm.

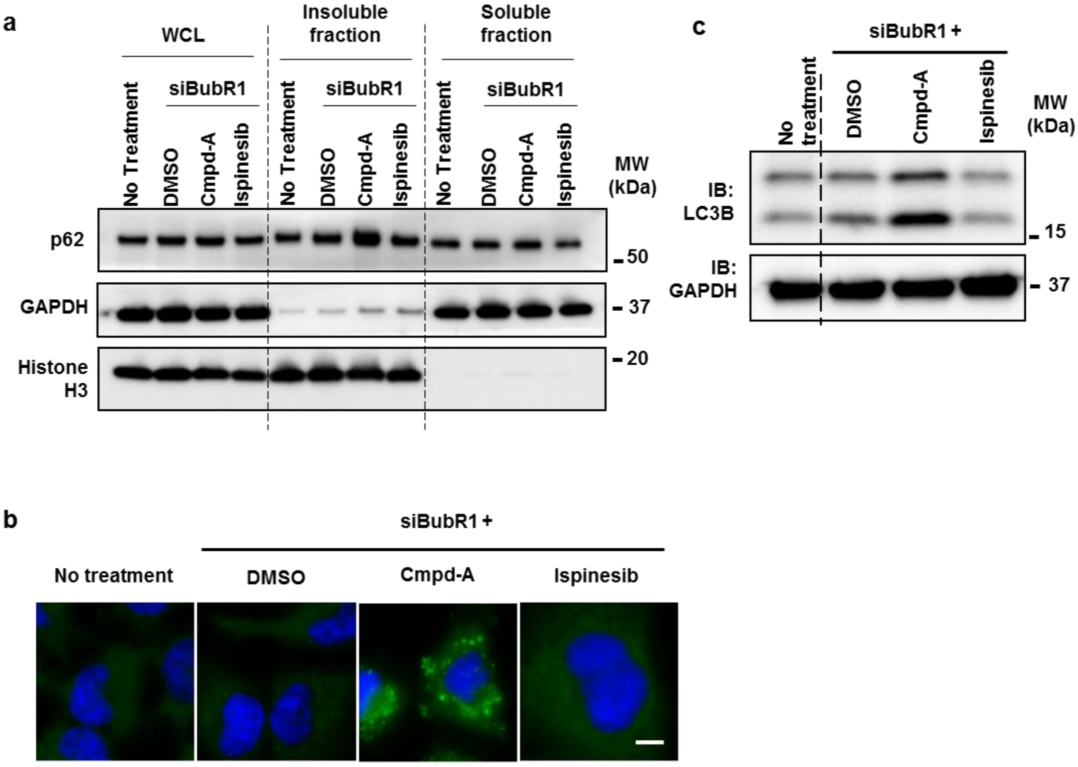

a
WCL
Insoluble fraction
Soluble fraction
siBubR1
siBubR1
siBubR1
No Treatment
DMSO
Cmpd-A
Ispinesib
No Treatment
DMSO
Cmpd-A
Ispinesib
No Treatment
DMSO
Cmpd-A
Ispinesib
MW (kDa)
p62
GAPDH
Histone H3
50
37
20
b
siBubR1 +
No treatment
DMSO
Cmpd-A
Ispinesib
c
siBubR1 +
No treatment
DMSO
Cmpd-A
Ispinesib
MW (kDa)
IB: LC3B
IB: GAPDH
15
37

Supplementary Figure 14. Proteotoxic stress in siBubR1+Cmpd-A or siBubR1+Ispinesib cells (related to main figure 6)

a. Protein accumulation of p62 in insoluble fraction after Compound-A treatment. The siBubR1 HeLa cells were treated with 200 nM Compound-A or 10 nM Ispinesib for 48 h. Cell lysates with RIPA buffer were fractionated to soluble and insoluble proteins. Immunoblotting was performed as described in Fig. 1a. **b.** Immunofluorescence of p62 in siBubR1+DMSO, siBubR1+Cmpd-A-, and siBubR1+ispinesib-treated HeLa cells. White bars indicate 10 μm. **c.** Protein accumulation of LC3B after Compound-A treatment. The isBubR1 cells were treated with 200 nM Compound-A or 10 nM Ispinesib for 48 h. Immunoblotting of LC3B was performed as described in Fig. 1a.

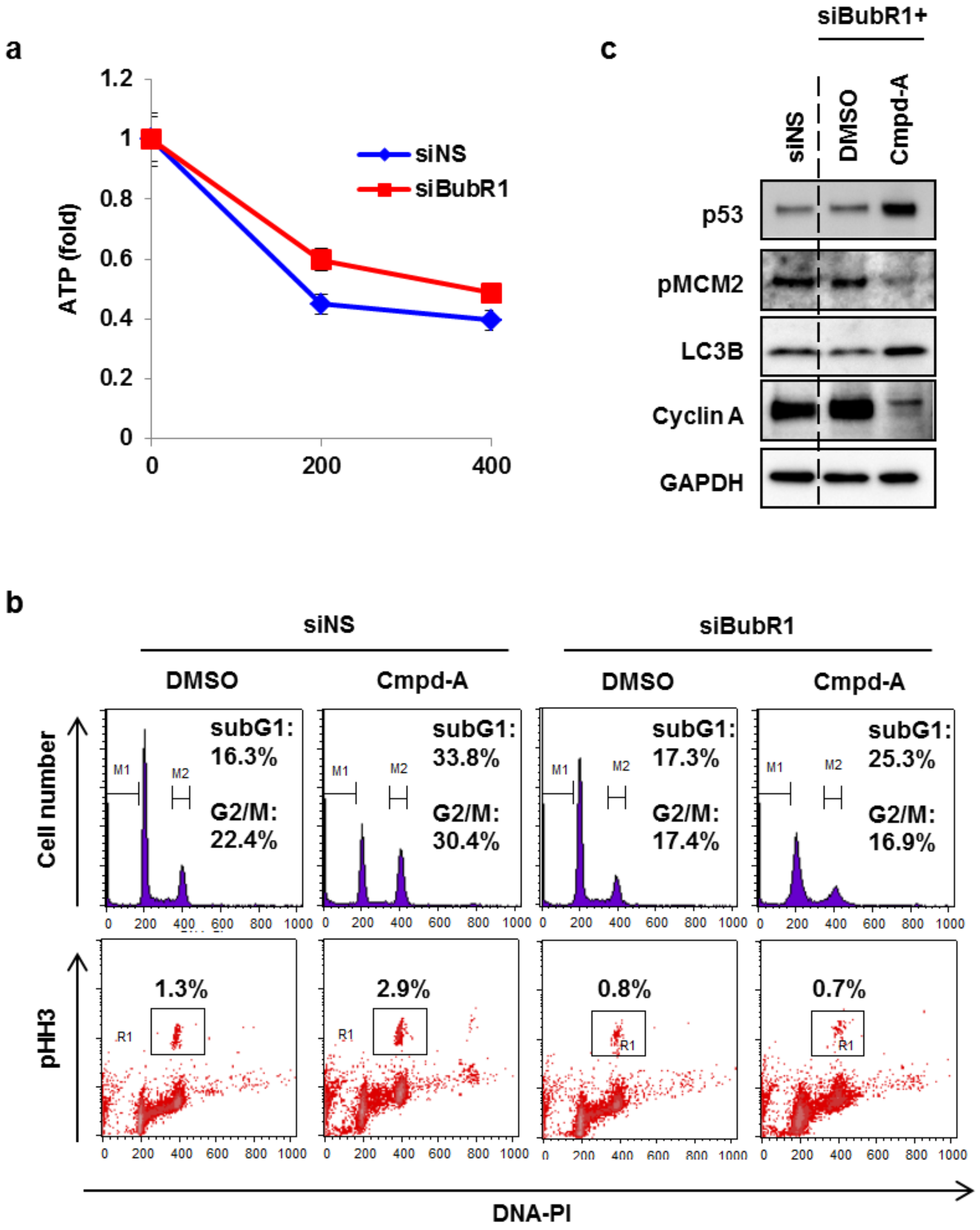

a
ATP (fold)
1.2
1
0.8
0.6
0.4
0.2
0
0
200
400
siNS
siBubR1
c
siBubR1+
siNS
DMSO
Cmpd-A
p53
pMCM2
LC3B
Cyclin A
GAPDH
b
siNS
siBubR1
DMSO
Cmpd-A
DMSO
Cmpd-A
Cell number
subG1: 16.3%
G2/M: 22.4%
subG1: 33.8%
G2/M: 30.4%
subG1: 17.3%
G2/M: 17.4%
subG1: 25.3%
G2/M: 16.9%
M1
M2
pHH3
1.3%
2.9%
0.8%
0.7%
R1
0 200 400 600 800 1000
DNA-PI

Supplementary Figure 15. Effects of siBubR1+Cmpd-A on anti-proliferation and p53 elevation in MCF10A cells. (related to main figure 6)

a. Anti-proliferative effect of Cmpd-A in siNS and siBubR1 MCF10A. Twenty-four hours after siRNA transfection, MCF10A cells were treated with or without Cmpd-A (200 nM). Cells were collected on day4 after drug treatment for the ATP assay. Relative ATP levels were calculated on the basis of luminescence in comparison with the luminescence value of 0 nM treatment for each. The line plots represent mean ± SD (n=3). **b.** Effect of Cmpd-A on mitotic arrest in siNS and siBubR1-transfected MCF10A. Twenty-four hours after siRNA transfection, MCF10A cells were treated with or without Cmpd-A (200 nM). Cells were collected on 24 hours after drug treatment for FACS assay. **c.** Immunoblotting of p53, pMCM2, LC3B, and Cyclin A in siBubR1+Cmpd-A MCF10A. Twenty-four hours after siRNA transfection, MCF10A cells were treated with or without Cmpd-A (200 nM) for 4 days.

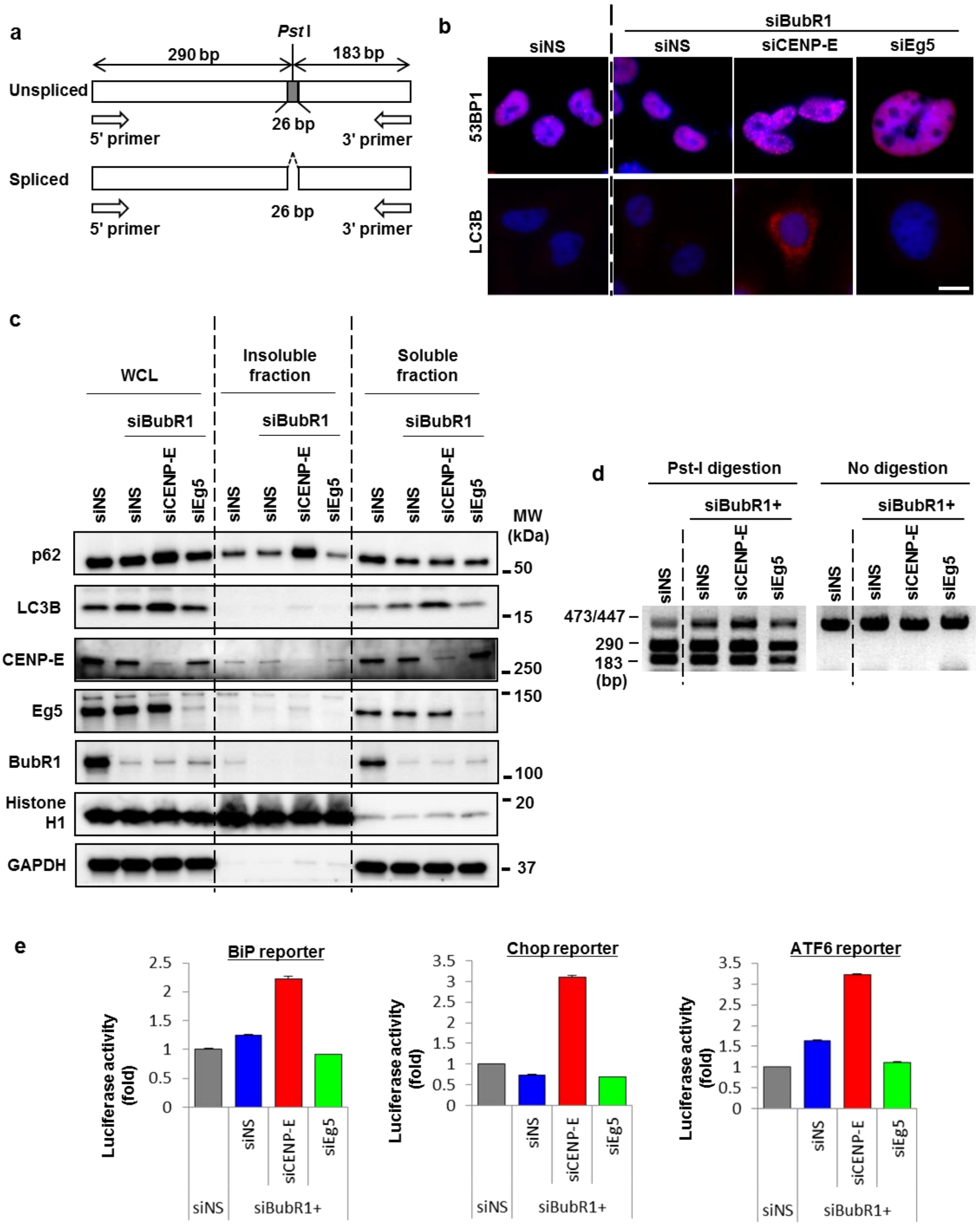
a
Pst I
290 bp
183 bp
Unspliced
26 bp
5' primer
3' primer
Spliced
26 bp
5' primer
3' primer
b
siBubR1
siNS
siNS
siCENP-E
siEg5
53BP1
LC3B
c
WCL
Insoluble fraction
Soluble fraction
siBubR1
siNS
siCENP-E
siEg5
MW (kDa)
p62
LC3B
CENP-E
Eg5
BubR1
Histone H1
GAPDH
50
15
250
150
100
20
37
d
Pst-I digestion
No digestion
siBubR1+
siNS
siCENP-E
siEg5
473/447
290
183
(bp)
e
BiP reporter
Chop reporter
ATF6 reporter
Luciferase activity (fold)
siNS
siCENP-E
siEg5
siBubR1+

Supplementary Figure 16. Proteotoxic stress in siBubR1+siCENP-E or siBubR1+siEg5 cells. (related to main figure 6)

a. Schematic of XBP1 splicing assay. **b.** Immunofluorescence of 53BP1 and LC3B in siCENP-E+siBubR1- and siEg5+siBubR1-transfected HeLa cells. The cells were treated with the indicated siRNAs for 72 hours. Immunofluorescence was performed as described in Fig. 1f and 6C. White bar indicates 20 μm. **c.** p62 induction in insoluble fraction and LC3BII formation. HeLa cells were treated with the indicated siRNAs for 72 h. Cell lysates with RIPA buffer were fractionated to soluble and insoluble proteins. Immunoblotting was performed as described in Fig. 1a. **d.** XBP1 splicing assay. HeLa cells were treated with the indicated siRNAs for 48 h. cDNA was treated with (left) or without (right) Pst-I. The undigested upper band (473/447 bp) and the digested lower bands (290 and 183 bp) represent spliced and unspliced XBP1, respectively. **e.** Transcriptional reporter activities in siCENP-E+siBubR1 or siEg5+siBubR1 HeLa cells. Reporter assays were performed as described in Fig. 6c. Hela cells were treated with the indicated siRNAs for 72 hours. The data represent mean ± SD (n=3).

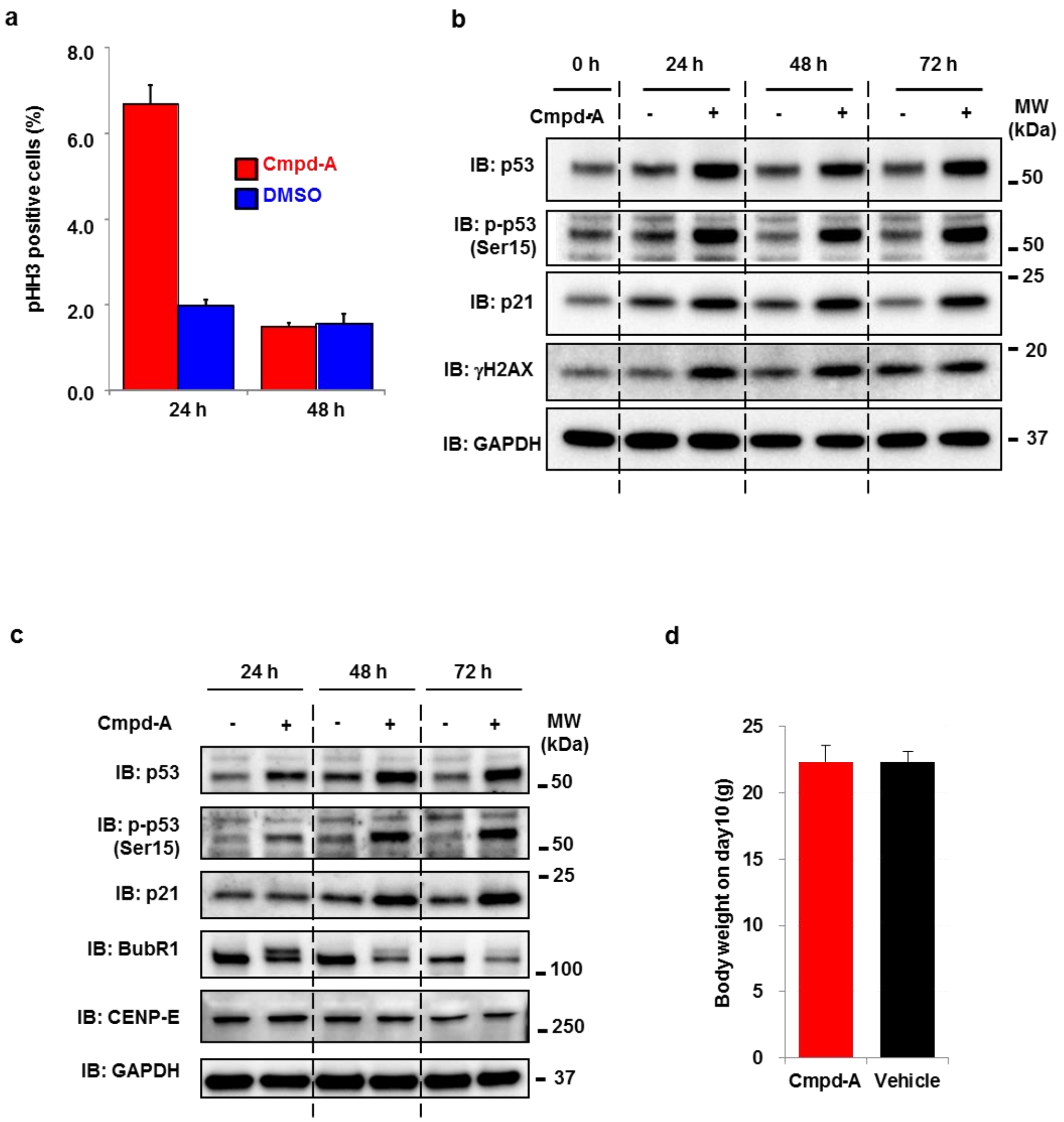

a
pHH3 positive cells (%)
8.0
6.0
4.0
2.0
0.0
Cmpd-A
DMSO
24 h
48 h
b
0 h
24 h
48 h
72 h
Cmpd-A
MW (kDa)
IB: p53
IB: p-p53 (Ser15)
IB: p21
IB: γH2AX
IB: GAPDH
50
50
25
20
37
c
24 h
48 h
72 h
Cmpd-A
MW (kDa)
IB: p53
IB: p-p53 (Ser15)
IB: p21
IB: BubR1
IB: CENP-E
IB: GAPDH
50
50
25
100
250
37
d
Body weight on day10 (g)
25
20
15
10
5
0
Cmpd-A
Vehicle

Supplementary Figure 17. Accumulation of p53 protein and anti-tumor activity by Cmpd-A treatment in the SAC-attenuated cells. (related to main figure 8)

a. Mitotic slippage in Caki-1 cells following Cmpd-A treatment. The cells were collected 24 and 48 h after Cmpd-A treatment. The data represent mean ± SD (n=3). **b.** Accumulation of p53 protein by Cmpd-A treatment in Caki-1 cells. Cells were collected 0, 24, 48 or 72 h after Compound-A treatment. **c.** Accumulation of p53 protein in Cmpd-A U87MG cells. Cells were collected 24, 48 or 72 h after Cmpd-A treatment. **d.** Body weight of nude mice treated with Cmpd-A in the Caki-1-xenograft models. Body weight comparison on day 8 after administration is shown (red bar: Cmpd-A treatment, black bar: vehicle control). The dats represent mean ± SD (n=5).

a

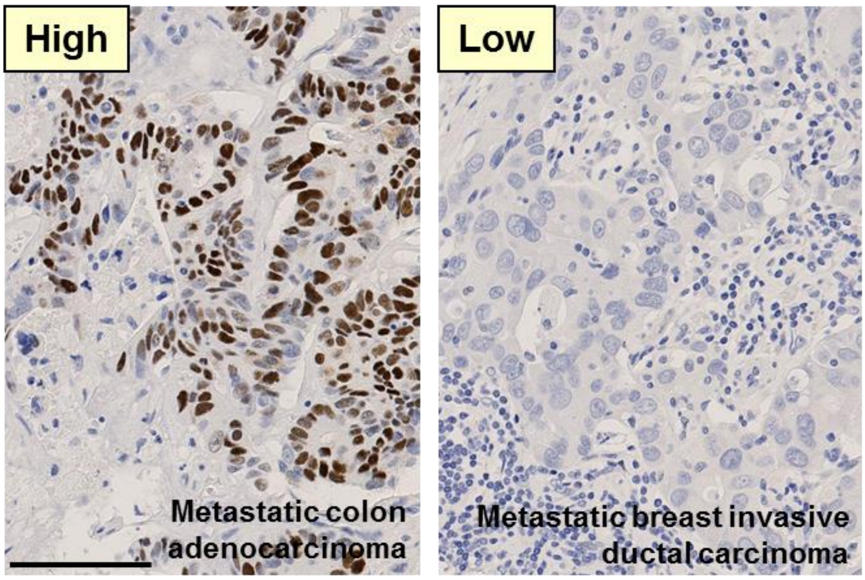
High
Low
Metastatic colon adenocarcinoma
Metastatic breast invasive ductal carcinoma

b

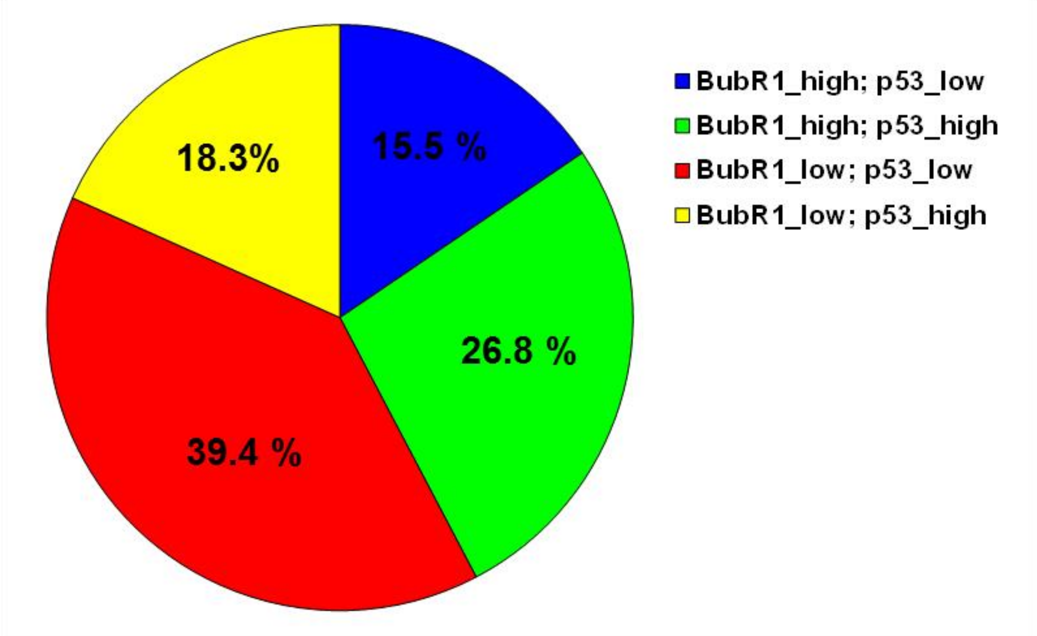
15.5 %
26.8 %
39.4 %
18.3%
BubR1_high; p53_low
BubR1_high; p53_high
BubR1_low; p53_low
BubR1_low; p53_high

Supplementary Figure 18. Immunohistochemistry of p53 in primary tumor tissues (related to main figure 9)

a. Immunohistochemistry of p53 in primary tumors. The representative images of p53 high expression (left) and low expression (right) are shown. Black bar indicates 100 μm.

b. Summary of expression profile of BubR1 and p53 in 71 primary human tumors. Tissue arrays of multiple tumors and pancreas tumors were stained with antibody against p53 and BubR1. Blue, green, red and yellow indicate percentage of tumors with high BubR1; low p53, high BubR1; high p53, low BubR1; low p53, and BubR1; low p53, respectively.

Fig. 1a

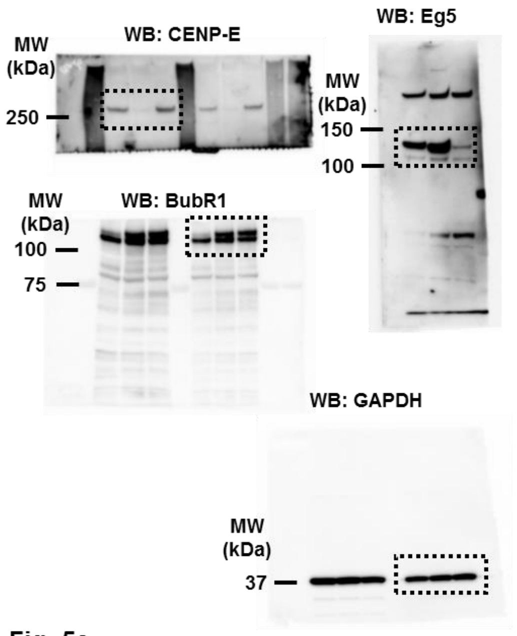

Fig. 4e

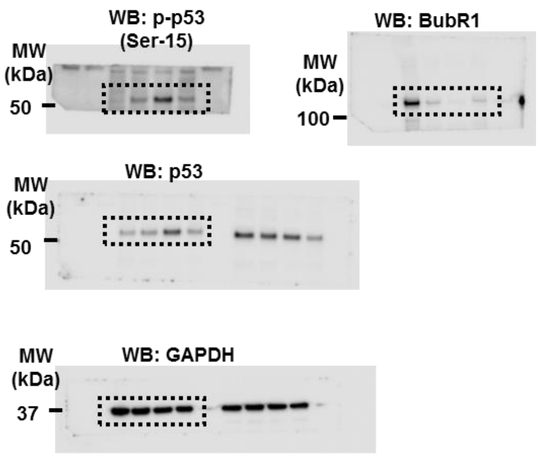

Fig. 5e

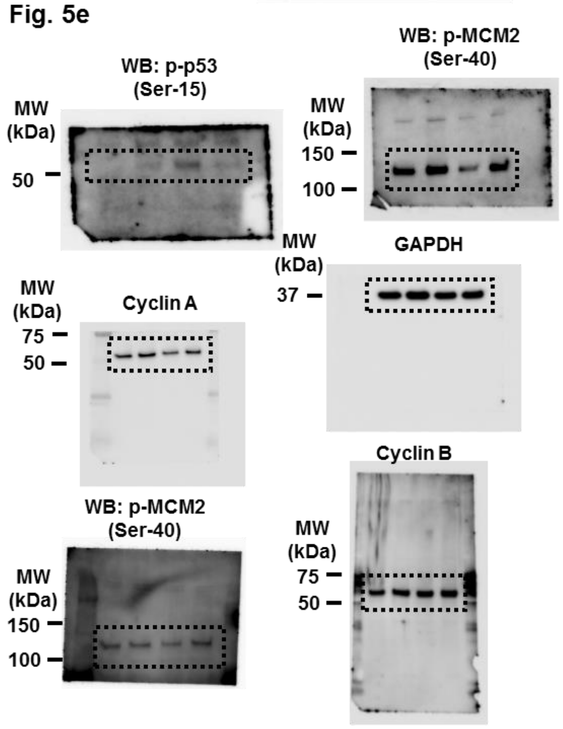

Supplementary Figure 19. Original images of immunoblots for Fig 1a, 4e, and 5e (related to main figure 1, 4, and 5)

Fig. 8b

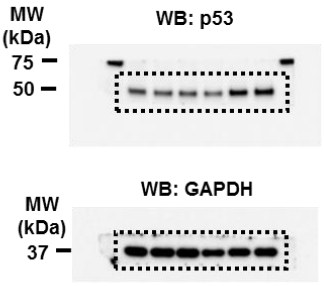
MW
(kDa)
WB: p53
75
50
WB: GAPDH
37

Fig. 8c

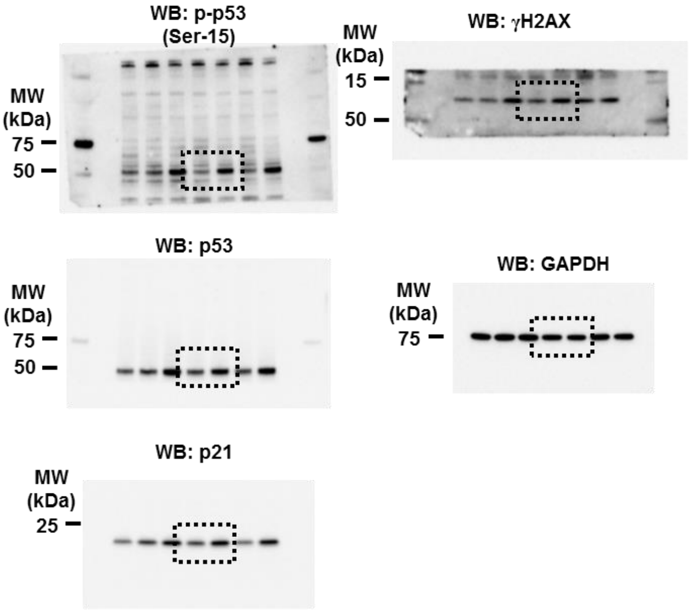
WB: p-p53
(Ser-15)
MW
(kDa)
75
50
WB: γH2AX
15
50
WB: p53
75
50
WB: GAPDH
75
WB: p21
25

Supplementary Figure 20. Original images of immunoblots for Fig. 8b and 8c (related to main figure 8)

Supplementary Table 1. Product ID and target sequence of siRNA

Gene Symbol	siRNA ID	Sense siRNA Sequence
CENP-E	D-003252-05	GAAGACAGCUCAAAUAAUAAU
	D-003252-06	CAACAAAGCUACUAAAUCAUU
	D-003252-07	GGAAAGAAGUGCUACCAUAUU
	D-003252-08	GGAAAGAAAUGACACAGUUUU
Eg5	D-003317-05	GCAGAAAUCUAAGGAUAUAUU
	D-003317-06	CAACAAGGAUGAAGUCUAUUU
	D-003317-07	CAGCAGAAAUCUAAGGAUAUU
	D-003317-08	CUAGAUGGCUUUCUCAGUAUU
BubR1	D-004101-01	GGAAGAAGAUCUAGAUGUAUU
	D-004101-02	CAAGAUGGCUGUAUUGUUUUU
	D-004101-03	CAAUACAGCUUCACUGAUAUU
	D-004101-04	GGAACAACCUCAUUCUAAAUU
TP53	D-003329-05	GAGGUUGGCUCUGACUGUA
	D-003329-07	GCACAGAGGAAGAGAAUCU
	D-003329-08	GAAGAAACCACUGGAUGGA
	D-003329-026	GCUUCGAGAUGUUCCGAGA
MAD2L1	11361	GGAUGAAAUCCGUUCAGUGtt
	11541	GGUCAUCUAUAGUUGAUAUtt
	11455	GGAUGACAUGAGGAAAAUAtt
BUB1	509	GGUUAUUUCAGACACGCCUtt
	147345	GCAGGUUGUUAUGUAUUGCtt
	510	GGCAAAAGCUGAAGAAAGUtt
TTK	392	GGAGGAAAAGAAGAAUUUAtt
	394	GGACUGGUUGAGUUUGUUGtt
	393	GGAAAAGAAGAAUUUAUCAtt
MAD1L1	13441	GGAAGCCAAUCAGAAAAUCtt
	121448	GCGAUUGUGAAGAACAUGAtt
	13280	GGCUCUGGACUGGAUAUUUtt
BUB3	15162	GGACUAGAUCAUCAAUUGAtt
	137638	GCAGGGUUAUGUAUUAAGCtt
	137637	GCCUGAAAAGGUAUAUACCtt

Supplementary Table 2. Product ID of TaqMan probe and primer set

Gene Symbol	TaqMan(R) Gene Expression Assay ID
BubR1	Hs00176169_m1
MAD2L1	Hs00829154_g1
BUB1	Hs00177821_m1
TTK	Hs00177412_m1
MAD1L1	Hs00269119_m1
BUB3	Hs00190920_m1

Supplementary Methods

CENP-E Enzyme Assay

An ATPase assay was used for determining the human kinesin activity. The CENP-E motor domain was purchased from Cytoskeleton, Inc. (Denver, CO, USA). The ATPase assay for determining the human CENP-E activity was performed using 62.5 ng/mL CENP-E motor domain, 22 μg/mL Microtubule (Cytoskeleton), and 25 μM ATP. Reactions were performed in 6 μL of reaction buffer [20 mM piperazine-*N*,*N*'-bis(2-ethanesulfonic acid) (PIPES)-KOH, pH 6.8, 3.0 mM $MgCl_2$, 3.0 mM KCl, 1.0 mM ethylene glycol tetraacetic acid (EGTA), 1.0 mM dithiothreitol, 0.01% w/v Brij-35, 0.2% w/v bovine serum albumin (BSA)] for 60 min at room temperature. The amount of ADP produced during the ATPase reaction was determined with the ADP-Glo kit (Promega). The luminescence was measured using an Envision plate reader (PerkinElmer, Inc., Waltham, MA, USA).

Cell cultures

HeLa, PANC1, and HT29 cells were cultivated in Dulbecco's modified Eagle's medium (DMEM) supplemented with 10% FBS. DU145 and U-87MG cells were cultivated in modified Eagle's medium (MEM) supplemented with 10% FBS.

NCI-H460 and COLO205 cells were cultivated in RPMI1640 medium supplemented with 10% FBS. A2780 cells were cultivated in Ham's F12 nutrient mixture supplemented with 10% FBS. HCT116, SK-BR-3, and Caki-1 cells were cultivated in McCoy's 5a medium supplemented with 10% FBS. MCF10A cells were cultivated in MEGM medium supplemented with 10% FBS. P53-wild and p53-null HCT116 cells of X-MAN Isogenic Cell Line were purchased from Horizon Discovery Group plc. (Cambridge, UK). All other cell lines were purchased from American Type Culture Collection (ATCC; Manassas, VA, USA)

Transfection of siRNA oligonucleotides

Pools of four (SMART pools, Dharmacon, Lafayette, CO, USA) or 3 (Ambion, Austin, TX, USA) predesigned siRNA oligos per gene of interest were tested. Three individual siRNAs from Ambion were pooled at equal molarity. siRNA oligonucleotides targeting CENP-E, Eg5 BubR1, MKLP2, Aurora-B, and survivin were obtained from Dharmacon, and siRNA oligonucleotides targeting INCENP, Mad2, Bub1, TTK, Mad1, and Bub3 were obtained from Ambion (Supplementary Table 1). The siTrio negative control (B-Bridge International, Inc., Mountain View, CA, USA) was used as a non-silencing (NS) siRNA (siNS).

Fifty nanomoles of pooled siRNA per gene were used for single knockdowns. Twenty-five nanomoles of pooled siRNA per gene were used for double (total siRNA concentration, 50 nM) or triple (total siRNA concentration, 75 nM) knockdowns. siRNA transfection was performed as described previously[1]. Transfection of siRNA oligonucleotides was performed with Dharmafect (Dharmacon) in 6-well plates according to the manufacturer's specifications.

RNA preparation and TaqMan quantitative RT-PCR analysis

Total RNA was extracted using the RNeasy Miniprep kit (Qiagen). cDNAs were synthesized from 500 ng of total RNA using the TaqMan Reverse Transcription Reagent kit (Applied Biosystems, Foster City, CA, USA). Real-time PCR was performed using an ABI PRISM 7900 instrument according to the manufacturer's protocol (Applied Biosystems). The 6-carboxyfluorescein (FAM) fluorescence released from each sample was measured as a function of the PCR cycle number (Ct) using the ABI PRISM 7900 instrument. The gene expression was calculated by the comparative Ct method[2]. The expression ratios of the indicated genes were quantified by the GAPDH expression in each cell line. The TaqMan probe product ID's are provided in Supplementary Table 2.

Cell-cycle synchronization

For cell-cycle synchronization at the G1/S phase, a dT block was prepared. Cells were treated with 2 mM thymidine (Sigma-Aldrich, St. Louis, MO, USA) for 16 h (first block), followed by incubation in thymidine-free medium for 8 hours. The cells were treated again with 2 mM thymidine for 16 h (second block). The cells were collected at 0, 2, 4, 6, 8, 10, 12, and 14 h after release from the second block.

For cell-cycle synchronization at the M phase, the cells were treated with 100 ng/mL nocodazole for 16 h.

Immunoblotting

Immunoblotting was performed as described previously[1]. The following antibodies were used at a concentration of 0.1-0.5 μg/ml: anti-CENP-E (1/1,000 dilution, sc22790; Santa Cruz Biotechnology), anti-p53 (1/1,000 dilution, sc126; Santa Cruz Biotechnology), anti-γH2AX (1/1,000 dilution, 2577; Cell Signaling Technology), anti-phospho-p53 (1/1,000 dilution, 9284; Cell Signaling Technology), anti-p21 (1/1,000 dilution, 2947; Cell Signaling Technology), anti-BubR1 (1/1,000 dilution, 612503; MD Transduction), anti-Eg5 (1/1,000 dilution, Ab37814; Abcam), anti-pHH3

(1/1,000 dilution, 06570; Upstate Biotechnology), anti-α-tubulin (1/1,000 dilution, T9026; Sigma-Aldrich), anti-Cyclin B1 (1/1,000 dilution, sc752; Santa Cruz Biotechnology), anti-BiP (1/1,000 dilution, 3183; Cell Signaling Technology), anti-p62 (1/1,000 dilution, sc28359; Santa Cruz Biotechnology), anti-ubiquitin (1/1,000 dilution, 3936; Cell Signaling Technology), and anti-GAPDH (1/10,000 dilution, MAB374; Chemicon). Immunoblotted proteins were visualized by chemiluminescence. Uncropped immunoblots are shown in Supplementary Fig. 19 and 20.

GeneChip microarray analysis

Total RNAs were extracted using the RNeasy Miniprep kit (Qiagen, Valencia, CA, USA) according to the manufacturer’s protocol. Preparation of the cDNAs and cRNAs, hybridization, and microarray scanning were performed according to the manufacturer’s protocols (Affymetrix Inc., CA, USA). The biotinylated cRNAs were hybridized to Affymetrix U133 Plus 2 human genome arrays. The captured signals were normalized to the median expression level using the GeneSpring software package (Agilent Technology), and the normalized data were filtered by present/absent calls and the expression level. A list of 343 p53-regulated genes (831 transcripts) was defined in the Ingenuity Pathways Analysis program using a query of genes for which the

expression is directly regulated by p53. (Ingenuity, Mountain View, CA, USA, http://www.ingenuity.com/products/pathways_analysis.html).

The microarray data have been deposited in NCBI's Gene Expression Omnibus, and are accessible through GEO Series accession number GSE67905 (http://www.ncbi.nlm.nih.gov/geo/query/acc.cgi?acc=GSE67905).

Immunohistochemistry

Endogenous peroxidases were quenched by addition of 3% H_2O_2. Antigen retrieval was performed by heating the samples in 10 mM citrate buffer (pH 6.0). The sections were incubated with rabbit anti-p53 antibody (1/200 dilution, #2527, Cell Signaling) and rabbit anti-BubR1 antibody (1/200 dilution, #5421 Cell Signaling) at a concentration of 1-2 μg/ml. The avidin-biotin-peroxidase complex system (Histofine Simple Stain Mouse MAX PO, Nichirei Bioscience) was used for color development with diaminobenzidine tetrahydrochloride. Tissue arrays of multiple tumors (Z7020082, Lot# B508080) and pancreas tumor (Z7020090, Lot# B507121) were purchased from BioChain Institute, Inc.

Reporter plasmid construction

The XBP1-Luc plasmid was generated by cloning the human XBP1 splicing region, nucleotides 410-633 relative to the start of the transcription of the human XBP1 cDNA, into the pGL4.13 vector (Promega). The pGRP78 (BiP)-Luc plasmid was created by cloning the human GRP78 promoter region nucleotides -160 to +7 into the pGL3 vector (Promega). The GADD153 (CHOP)-Luc plasmid was generated by cloning the human sequence 500-bp upstream of the transcriptional start site into the pGL3 vector (Promega). The ATF6-Luc plasmid was created by cloning 5 tandem repeats of a stress-activated sequence motif capable of binding ATF6, CTCGAGACAGGTGCTGACGTGGCATTC, into the pGL3 vector (Promega).

Luciferase assay

The reporter plasmids were transfected into the BubR1-knockdown HeLa cells with the internal control plasmid pGL4.74 (Promega). Eight hours after the plasmid transfection, the cells were treated with Compound-A (200 nM), Ispinesib (10 nM), or DMSO for 72 h. The luciferase activity of these cells was measured using the Dual-Luciferase Reporter Assay System (Promega, E1910), with a microplate reader, according to the manufacturer’s protocol. The siNS-HeLa cells treated with DMSO were used as a negative control, and the relative luciferase activities were calculated

based on chemical luminescence values in comparison with the values of the control (n = 3).

Electron microscopy

Electron microscopy was performed by Tokai Electron Microscopy, Inc. The samples were fixed with 2% paraformaldehyde, 2% glutaraldehyde in 0.1 M phosphate buffer (pH 7.4), followed by post fixation with 2% osmium tetroxide. The fixed samples were dehydrated through a series of ethanol (50, 70, 90 and 100%), and then transferred to a resin (Quetol-812; Nisshin EM CO., Tokyo, Japan) and polymerized at 60ºC.

The blocks were ultra-thin sectioned at 70 nm using a ultramicrotome (ULTRACUT UCT; Leica), and stained with 2% uranyl acetate followed by Lead stain solution (Sigma-Aldrich Co.). The samples were onserved by a transmission electron microscope (JEM-1400Plus; JEOL Ltd.,) at an acceleration voltage of 80 kV. Digital images (2048X2048 pixels) were taken with a CCD camera (VRLRTA; Olympus Soft Imaging Solution GmbH).

The neutral Comet Assay

The neutral comet assay was performed using CometAssay Kit according to the

manufacturer's protocol (Catalogue# 4250-050-K, TREVIGEN, MD, USA). Briefly, The 1×10^3 HeLa cells with the indicated siRNAs and compounds were mixed with 50 µl LNAgarose at 37 ºC and layered onto CometSlide. Slides were plced in the dark at 4°C until the agarose was set, and then immersed in Lysis Solution for 1 hour at 4 ºC. After the slides were immersed in Neutral Electrophoresis Buffer for 30 minutes at 4 ºC, the samples were applied to electrophoresis at 25 V for 30 minutes. The samples were fixed with 70% ethanol for 30 minutes at room temperature, and stained with CYBR GOLD. The microscopic images of DNA tails were analyzed by AxioVision (Carl Zeiss).

XBP1 Splicing Assay

The XBP-1 splicing assay was performed as described previously[3]. Briefly, the total RNA was extracted from cells, followed by one-step reverse transcription PCR. A 473 bp-cDNA product encompassing the splicing region (26 bp) was amplified using the indicated primer pairs, and then the PCR products were treated with *Pst-I* for 2 hours at 37 ºC to digest unspliced products containing *Pst-I* site in 26 bp-splicing region. The following primer pairs were used:

Sense primer, 5′ -AAACAGAGTAGCAGTCCAGACTGC-3′

Antisense primer, 5′ -TCCTTCTGGGTAGACCTCTGGGAG-3′

Synthetic Protocol for Compound-A[4]

Preparation of Compound 2

To a solution of 2,2,6,6-tetramethylpiperidine (980 mg) in tetrahydrofuran (THF) (6.5 mL) was slowly added a solution of *n*-butyllithium (*n*-BuLi) in hexanes (1.6 M, 4.0 mL) at –50 ºC under nitrogen atmosphere. The mixture was stirred at –50 ºC for 30 min. To the reaction mixture was slowly added a solution of 2-fluoro-6-(trifluoromethyl)benzonitrile (**1**) (1005 mg) in THF (3 mL) at –60 ºC. The mixture was stirred at –50 ºC for 30 min. To the reaction mixture was slowly added a solution of *N*,*N*-dimethylformamide (1170 mg) in THF (1 mL) at –50 ºC under nitrogen atmosphere. The mixture was stirred at –50 ºC for 15 min. It was then warmed up to –10 ºC and stirred for 20 min. To the mixture were added acetic acid (1 mL) and water. The resulting mixture was extracted with ethyl acetate (EtOAc). The organic layer was washed with saturated aqueous $NaHCO_3$ solution and brine, successively, and dried over anhydrous Na_2SO_4. The insoluble was filtered off and the filtrate was evaporated in vacuo to give 2-fluoro-3-formyl-6-(trifluoromethyl)benzonitrile (**2**) (1130 mg) as brown oil: ^{1}H NMR (300 MHz, $CDCl_3$) δ 7.77 (1H, d, J = 8.1 Hz), 8.25 (1H, t, J = 7.4 Hz),

10.42 (1H, s). MS (ESI): undetected.

Preparation of Compound 3

To a solution of **2** (1090 mg) in dimethylsulfoxide (DMSO) (13 mL) was added sodium methanesulphinate (630 mg) at room temperature. The reaction mixture was stirred at room temperature for 2.5 h. It was then diluted with EtOAc (30 mL) and the resulting suspension was washed with water (30 mL). The aqueous layer was extracted with EtOAc. The combined organic layer was washed with water and brine, successively, and dried over anhydrous Na_2SO_4. The insoluble was filtered off and the filtrate was evaporated in vacuo. The obtained solid was washed with a mixed solvent of EtOAc and hexanes to give 3-formyl-2-(methylsulfonyl)-6-(trifluoromethyl)benzonitrile (**3**) (890 mg) as brown solid: ^{1}H NMR (300 MHz, $CDCl_3$) δ 3.51 (3H, s), 8.12–8.17 (1H, m), 8.19–8.23 (1H, m), 10.73 (1H, s). MS (ESI−): 275.8 $[M-H]^-$.

Preparation of Compound 4

To a solution of **3** (570 mg) in THF (25 mL) was added *N*,*N*-dimethylethane-1,2-diamine (290 μL) at room temperature. The mixture was stirred

at room temperature for 2 h. To the mixture was slowly added a solution of lithium *N*,*N*-diisopropylamide (LDA) in hexanes/THF (1.11 M, 3720 μL) at 0 ºC under nitrogen atmosphere. The mixture was stirred at 0 ºC for 50 min. It was then diluted with EtOAc (30 mL), and the resulting suspension was washed with water (30 mL). The aqueous layer was extracted with EtOAc. The combined organic layer was washed with brine and dried over anhydrous Na_2SO_4. The insoluble was filtered off and the filtrate was evaporated in vacuo. The obtained crude material was purified by aminopropyl-coated silica gel chromatography (NH, (Fuji Silysia), eluent: EtOAc/*n*-hexane). Desired fraction was evaporated in vacuo to give (±)-3-{[2-(dimethylamino)ethyl]amino}-6-(trifluoromethyl)-2,3-dihydro-1-benzothiophene-7-carbonitrile 1,1-dioxide (**4**) (116 mg) as yellow oil: ^{1}H NMR (300 MHz, $CDCl_3$) δ 2.19–2.24 (6H, m), 2.36–2.55 (2H, m), 2.65–2.80 (2H, m), 3.49 (1H, dd, J = 13.4, 5.9 Hz), 3.93 (1H, dd, J = 13.5, 7.2 Hz), 4.70–4.81 (1H, m), 7.99–8.05 (1H, m), 8.06–8.12 (1H, m). MS (ESI+): 348.2 $[M+H]^+$.

Preparation of Compound-A

To a solution of 3-(4-fluoro-3-methylphenyl)-5-methoxyimidazo[1,2-*a*] pyridine2-carboxylic acid (**5**) (CAS: 1355082-64-2, PCT Int. Appl. (2012)

WO2012008508A1) (120 mg) in *N*,*N*-dimethylformamide (DMF) (4 mL) were added **4** (116 mg), *O*-(7-azabenzotriazol-1-yl)-*N*,*N*,*N*',*N*'-tetramethyluronium hexafluorophosphate (HATU) (190 mg) and *N*,*N*-diisopropylethylamine (DIEA) (130 mg) at room temperature. The mixture was stirred at 50 ºC for 6 h. The mixture was diluted with EtOAc (20 mL) and the resulting suspension was washed with anhydrous aqueous $NaHCO_3$ solution (20 mL). The aqueous layer was extracted with EtOAc. The combined organic layer was washed with water and brine, successively, and dried over anhydrous Na_2SO_4. The insoluble was filtered off and the filtrate was concentrated in vacuo. The obtained crude material was purified by aminopropyl-coated silica gel chromatography (NH, (Fuji Silysia), eluent: EtOAc/*n*-hexane). Desired fraction was evaporated in vacuo. The obtained solid was washed with a mixed solvent of EtOAc and hexanes to give the racemic Compound-A (140 mg): ^{1}H NMR (300 MHz, $CDCl_3$) δ 2.07 (6H, s), 2.23–2.35 (3H, m), 2.37–2.64 (2H, m), 3.55–3.92 (6H, m), 4.18–4.81 (1H, m), 5.33–6.30 (2H, m), 6.90–7.24 (3H, m), 7.28 (2H, d, J = 4.2 Hz), 7.51–8.02 (2H, m). MS (ESI+): 630.3 $[M+H]^+$.

The racemic Compound-A (132 mg) was subjected to preparative supercritical fluid chromatography (SFC) (CHIRALCEL ODH (KC003), 20 mmID × 250 mmL (DAICEL Corporation), eluent: CO_2/2-propanol/acetonitrile = 800/100/100). Desired

fraction of the enantiomer with a higher retention time (slower) was collected and concentrated in vacuo to give (+)-*N*-[7-cyano-1,1-dioxido-6-(trifluoromethyl)-2,3-dihydro-1-benzothiophen-3-yl]-*N*-[2-(dimethylamino)ethyl]-3-(4-fluoro-3-methylphenyl)-5-methoxyimidazo[1,2-*a*]pyridine-2-carboxamide (Compound-A) (61 mg) as colorless amorphous solid: Optical purity 99.8% ee (CHIRALCEL ODH (LD029), 4.6 mmID × 150 mmL (DAICEL Corporation), eluent: CO_2/2-propanol/acetonitrile = 800/100/100). Specific optical rotation: [α]25. D. +74.3 (c 0.231, $CHCl_3$).^{13}C NMR (150 MHz, DMSO-d_6) δ 14.0 (d, J = 2.8 Hz), 44.8, 47.7, 53.3, 54.9, 56.5, 58.2, 90.2, 101.9 (q, J = 2.1 Hz), 109.3, 110.5, 113.4 (d, J = 22.0 Hz), 121.9 (q, J = 274.5 Hz), 122.3 (d, J = 17.6 Hz), 122.8, 126.2 (d, J = 3.3 Hz), 127.6, 130.0 (d, J = 8.3 Hz), 130.7, 131.2 (q, J = 4.5 Hz), 131.7 (q, J = 32.5 Hz), 133.7 (d, J = 5.5 Hz), 137.5, 141.3, 144.9, 146.0, 151.7, 160.2 (d, J = 243.7 Hz), 165.4.

Supplementary References

1. Ohashi, A., Zdzienicka, M.Z., Chen, J. & Couch, F.J. Fanconi anemia complementation group D2 (FANCD2) functions independently of BRCA2- and RAD51-associated homologous recombination in response to DNA damage. *The Journal of biological chemistry* **280**, 14877-14883 (2005).
2. Livak, K.J. & Schmittgen, T.D. Analysis of relative gene expression data using real-time quantitative PCR and the 2(-Delta Delta C(T)) Method. *Methods* **25**, 402-408 (2001).
3. Calfon, M. *et al.* IRE1 couples endoplasmic reticulum load to secretory capacity by processing the XBP-1 mRNA. *Nature* **415**, 92-96 (2002).
4. Hirayama, T., Banno, H., Okaniwa, M., Kakei, H. & Ishikawa, T. Heterocyclic compound. *PCT International Patent*, **WO2013/100018 A1**, 4 Jul 2013.
